# Supplementary material for: The miRNA Content of Bone Marrow-Derived Extracellular Vesicles Contributes to Protein Pathway Alterations Involved in Ionising Radiation-Induced Bystander Responses
Source: Int J Mol Sci. 2023 May 11;24(10):8607. doi: 10.3390/ijms24108607 (PMC10218377; doi:10.3390/ijms24108607)
Supplement: Supplementary file 1 [file ijms-24-08607-s001.zip › Supplementary Figure S1.pdf]

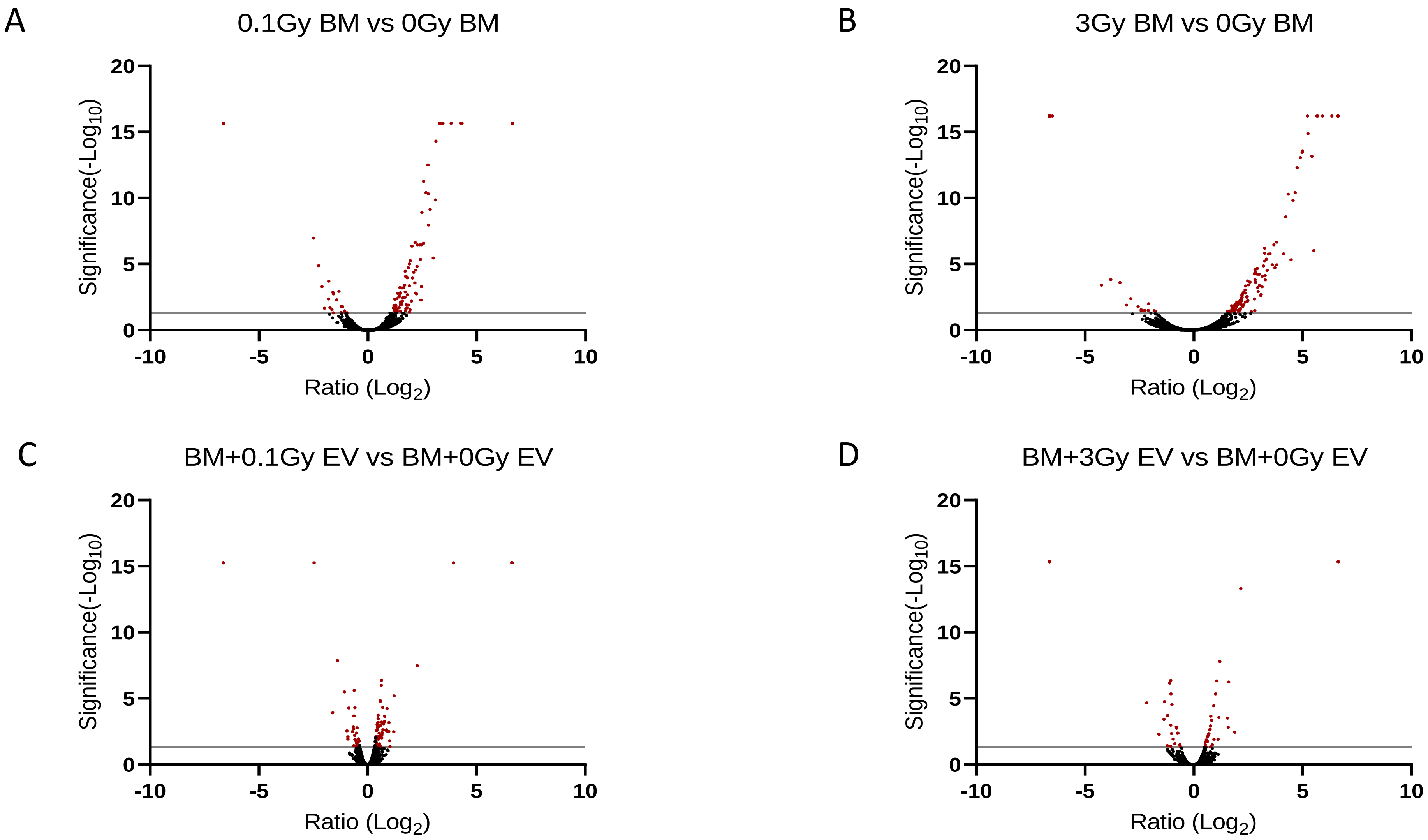

Supplementary Figure S1. Volcano plot of MS identified proteins. Proteins with high FDR confidence and at least 2 unique peptides are shown in the graphs. Red dots indicate the proteins that changed significantly compared to the control sample (0.05<p-value, Fold Change ≥1.33). The y-axis indicates the -log 10 of the p-values, the x-axis indicates the ratio of either directly irradiated (A: 0.1Gy, B: 3Gy) or EV-treated samples (C: BM+0.1Gy EV, D: BM+3Gy EV) and their controls.
