# Supplementary material for: The miRNA Content of Bone Marrow-Derived Extracellular Vesicles Contributes to Protein Pathway Alterations Involved in Ionising Radiation-Induced Bystander Responses
Source: Int J Mol Sci. 2023 May 11;24(10):8607. doi: 10.3390/ijms24108607 (PMC10218377; doi:10.3390/ijms24108607)
Supplement: Supplementary file 1 [file ijms-24-08607-s001.zip › Supplementary Figure S2.pdf]

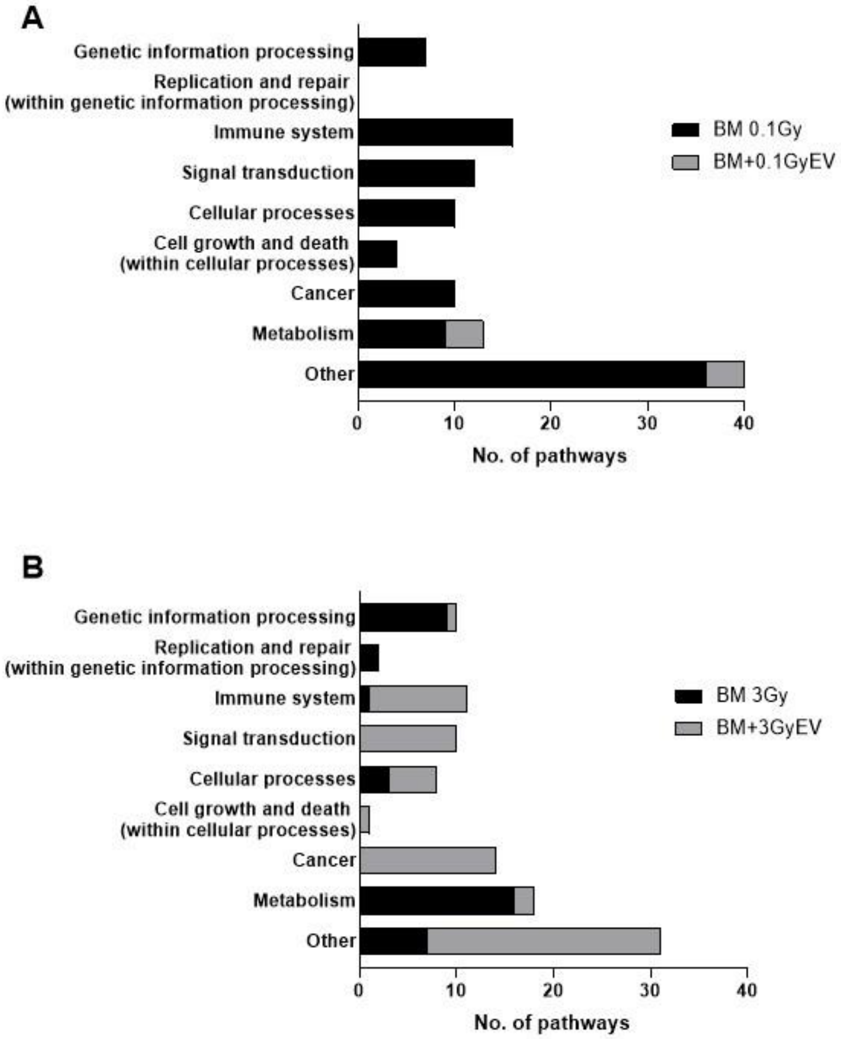

Supplementary Figure S2. Non-common pathways between directly irradiated and EV-treated samples. Significantly deregulated pathways were compared between directly irradiated and EV treated samples, and the distribution of non-common ones are presented (0.1Gy BM was compared with BM+0.1Gy EV, while 3Gy BM was compared to BM+3Gy EV).
