# Supplementary material for: The miRNA Content of Bone Marrow-Derived Extracellular Vesicles Contributes to Protein Pathway Alterations Involved in Ionising Radiation-Induced Bystander Responses
Source: Int J Mol Sci. 2023 May 11;24(10):8607. doi: 10.3390/ijms24108607 (PMC10218377; doi:10.3390/ijms24108607)
Supplement: Supplementary file 1 [file ijms-24-08607-s001.zip › Supplementary Table S1.pdf]

Supplementary Table S1. Differentially expressed miRNAs in bone marrow-derived extracellular vesicles from mice irradiated with 0.1Gy and 3Gy vs. controls. Analysis was performed with DIANA-mirExTra 2.0. Significant miRNAs had a p-value below 0.05. FDR: false discovery rate.

**0.1Gy**			
**Name**	**Fold Change**	**P-value**	**FDR**
mmu-miR-761	4.67	0.03	1.00
mmu-miR-129-5p	4.75	0.04	1.00
**3Gy**			
**Name**	**Fold Change**	**P-value**	**FDR**
mmu-miR-709	0.07	3.3e-4	0.08
mmu-miR-34b-5p	11.33	2.4e-3	0.20
mmu-miR-323-5p	13.67	2.6e-3	0.20
mmu-miR-761	8.75	4.9e-3	0.23
mmu-miR-291a-3p	8.25	5.3e-3	0.23
mmu-miR-323-3p	8.67	6.8e-3	0.23
mmu-miR-1946a	9.67	6.9e-3	0.23
mmu-miR-1933-5p	7.58	9.9e-3	0.29
mmu-miR-1898	7.75	0.01	0.29
mmu-miR-669g	6.75	0.01	0.29
mmu-miR-695	8.67	0.01	0.29
mmu-miR-706	0.15	0.02	0.30
mmu-miR-669h-5p	6.19	0.02	0.38
mmu-miR-338-5p	4.77	0.03	0.54
mmu-miR-466k	6.67	0.04	0.54
mmu-miR-1942	4.72	0.04	0.58
mmu-miR-129-5p	6.60	0.04	0.58
