# Supplementary material for: The miRNA Content of Bone Marrow-Derived Extracellular Vesicles Contributes to Protein Pathway Alterations Involved in Ionising Radiation-Induced Bystander Responses
Source: Int J Mol Sci. 2023 May 11;24(10):8607. doi: 10.3390/ijms24108607 (PMC10218377; doi:10.3390/ijms24108607)
Supplement: Supplementary file 1 [file ijms-24-08607-s001.zip › Supplementary Table S10.pdf]

Supplementary Table S10. MiRNA-protein interactions involved in the common pathways between differentially expressed miRNAs from bone marrow-derived extracellular vesicles from mice irradiated with 3Gy and deregulated proteins in bone marrow cells treated with bone marrow-derived extracellular vesicles of mice irradiated with 3Gy. Only those miRNA and protein interactions are presented, where both the miRNA and the protein could be linked to the certain common pathways. MiRNA-protein interactions were searched in Tarbase (validated interactions) and miRDB database (predicted interactions).

miRNA-protein interactions in common pathways			
**Interaction partners**		**Interaction type**	**Regulated pathway**
**Protein**	**miRNA**		
**Atg4b**	mmu-miR-706	validated	Non-small cell lung cancer
**Calu**	mmu-miR-761	predicted	Focal adhesion<br>Progesterone-mediated oocyte maturation<br>Protein processing in endoplasmic reticulum
**H2afv**	mmu-miR-706	validated	Cell cycle<br>Transcriptional misregulation in cancer
**Krt16**	mmu-miR-709	predicted	Chronic myeloid leukemia<br>Colorectal cancer<br>Focal adhesion<br>Glioma<br>Hepatitis B<br>Non-small cell lung cancer<br>Pancreatic cancer<br>Pathways in cancer<br>Prostate cancer<br>Proteoglycans in cancer
**Rtf1**	mmu-miR-761	predicted	Lysine degradation
**Snca**	mmu-miR-706	validated	Acute myeloid leukemia<br>Adherens junction<br>Cell cycle<br>Chronic myeloid leukemia<br>Colorectal cancer<br>Focal adhesion<br>Glioma<br>Hepatitis B<br>Non-small cell lung cancer
**Tgm2**	mmu-miR-761	predicted	Adherens junction<br>Cell cycle<br>Chronic myeloid leukemia<br>Colorectal cancer<br>Focal adhesion<br>Glioma<br>Hepatitis B<br>Non-small cell lung cancer<br>Transcriptional misregulation in cancer

			Pancreatic cancer<br>Pathways in cancer<br>Prostate cancer<br>Proteoglycans in cancer
**Ube2c**	mmu-miR-669g	validated	Cell cycle<br>Hepatitis B<br>Non-small cell lung cancer<br>Pancreatic cancer<br>Progesterone-mediated oocyte maturation<br>Prostate cancer<br>Protein processing in endoplasmic reticulum
	mmu-miR-669g and mmu-miR-129-5p	validated	Chronic myeloid leukemia
**Clic4**	mmu-miR-669g	predicted	Cell cycle<br>Chronic myeloid leukemia<br>Hepatitis B<br>Transcriptional misregulation in cancer<br>Prostate cancer<br>Proteoglycans in cancer
**Dnajb6**	mmu-miR-709	predicted	Cell cycle<br>Colorectal cancer<br>Hepatitis B<br>Non-small cell lung cancer<br>Pancreatic cancer<br>Pathways in cancer<br>Prostate cancer<br>Proteoglycans in cancer
**Eif2b1**	mmu-miR-1942	predicted	Adherens junction<br>Colorectal cancer
