# Supplementary material for: The miRNA Content of Bone Marrow-Derived Extracellular Vesicles Contributes to Protein Pathway Alterations Involved in Ionising Radiation-Induced Bystander Responses
Source: Int J Mol Sci. 2023 May 11;24(10):8607. doi: 10.3390/ijms24108607 (PMC10218377; doi:10.3390/ijms24108607)
Supplement: Supplementary file 1 [file ijms-24-08607-s001.zip › Supplementary Table S2.pdf]

Supplementary Table S2. Pathways regulated by differentially expressed miRNAs from the bone marrow-derived extracellular vesicles of irradiated mice. Pathway analysis was performed with Diana mirpath. The miRNAs differentially expressed in the bone marrow-derived extracellular vesicles of mice irradiated with 0.1Gy or 3Gy were used as an input. In the gene column, the number of genes that play a role in the given pathway and are under the control of input miRNAs is highlighted. In the miRNAs column, the number of miRNAs involved in the pathway was highlighted.

Pathways regulated by differentially expressed miRNAs from the bone marrow-derived extracellular vesicles of mice irradiated with 0.1Gy					
**KEGG pathway**	**KEGG pathway class**	**KEGG pathway subclass**	**p-value**	**genes**	**miRNAs**
TGF-beta signaling pathway	3. Environmental Information Processing- Signal transduction	3.2 Signal transduction	1,25E-06	4	2
Signaling pathways regulating pluripotency of stem cells	4. Cellular Processes	4.3 Cellular community - eukaryotes	2,48E-03	5	2
Primary bile acid biosynthesis	1. Metabolism	1.3 Lipid metabolism	1,19E-02	1	1
Pathways regulated by differentially expressed miRNAs from the bone marrow-derived extracellular vesicles of mice irradiated with 3Gy					
**Pathway name**	**KEGG pathway class**	**KEGG pathway subclass**	**p-value**	**genes**	**miRNAs**
Prion diseases	6.Diseases	6.7 Neurodegenerative disease	4,56E-17	4	3
Fatty acid biosynthesis	1. Metabolism	1.3 Lipid metabolism	3,30E-16	3	2
Lysine degradation	1. Metabolism	1.5 Amino acid metabolism	2,37E-06	15	5
Proteoglycans in cancer	6.Diseases-cancer	6.1 Cancer: overview	1,59E-04	38	7
Protein processing in endoplasmic reticulum	2. Genetic information processing	2.3 Folding, sorting and degradation	2,05E-04	42	9
Hippo signaling pathway	3. Environmental Information Processing- Signal transduction	3.2 Signal transduction	6,22E-04	28	11
Prostate cancer	6.Diseases-cancer	6.2 Cancer: specific types	1,15E-03	24	8
Focal adhesion	4. Cellular Processes	4.3 Cellular community - eukaryotes	1,66E-03	46	8
Adherens junction	4. Cellular Processes	4.3 Cellular community - eukaryotes	1,75E-03	18	6
Cell cycle	4. Cellular Processes	4.2 Cell growth and death	2,07E-03	26	8
Phosphatidylinositol signaling system	3. Environmental Information Processing- Signal transduction	3.2 Signal transduction	2,85E-03	16	5
mTOR signaling pathway	3. Environmental Information Processing- Signal transduction	3.2 Signal transduction	2,85E-03	19	9

Signaling pathways regulating pluripotency of stem cells	4. Cellular Processes	4.3 Cellular community - eukaryotes	2,85E-03	27	9
Hepatitis B	6.Diseases-Infectious	6.3 Infectious disease: viral	2,85E-03	27	8
Colorectal cancer	6.Diseases-cancer	6.2 Cancer: specific types	3,46E-03	17	5
TGF-beta signaling pathway	3. Environmental Information Processing- Signal transduction	3.2 Signal transduction	5,19E-03	17	7
Glioma	6.Diseases-cancer	6.2 Cancer: specific types	5,19E-03	15	6
FoxO signaling pathway	3. Environmental Information Processing- Signal transduction	3.2 Signal transduction	8,55E-03	27	7
Regulation of actin cytoskeleton	4. Cellular Processes	4.5 Cell motility	8,55E-03	40	7
Chronic myeloid leukemia	6.Diseases-cancer	6.2 Cancer: specific types	8,55E-03	19	6
Pathways in cancer	6.Diseases-cancer	6.1 Cancer: overview	1,18E-02	62	10
Progesterone-mediated oocyte maturation	5. Organismal Systems	5.2 Endocrine system	1,53E-02	22	8
Platelet activation	5. Organismal Systems-Immune system	5.1 Immune system	2,01E-02	26	7
Pancreatic cancer	6.Diseases-cancer	6.2 Cancer: specific types	2,37E-02	14	6
Acute myeloid leukemia	6.Diseases-cancer	6.2 Cancer: specific types	3,48E-02	14	5
MAPK signaling pathway	3. Environmental Information Processing- Signal transduction	3.2 Signal transduction	3,67E-02	45	8
Non-small cell lung cancer	6.Diseases-cancer	6.2 Cancer: specific types	4,02E-02	12	6
Inositol phosphate metabolism	1. Metabolism	1.1 Carbohydrate metabolism	4,06E-02	11	5
ErbB signaling pathway	3. Environmental Information Processing- Signal transduction	3.2 Signal transduction	4,06E-02	17	5
cAMP signaling pathway	3. Environmental Information Processing- Signal transduction	3.2 Signal transduction	4,06E-02	37	9
Circadian rhythm	5. Organismal Systems	5.10 Environmental adaptation	4,06E-02	8	3
Neurotrophin signaling pathway	5. Organismal Systems	5.6 Nervous system	4,06E-02	23	9
Transcriptional misregulation in cancer	6.Diseases-cancer	6.1 Cancer: overview	4,06E-02	32	11
Wnt signaling pathway	3. Environmental Information Processing- Signal transduction	3.2 Signal transduction	4,75E-02	28	6
