# Supplementary material for: The miRNA Content of Bone Marrow-Derived Extracellular Vesicles Contributes to Protein Pathway Alterations Involved in Ionising Radiation-Induced Bystander Responses
Source: Int J Mol Sci. 2023 May 11;24(10):8607. doi: 10.3390/ijms24108607 (PMC10218377; doi:10.3390/ijms24108607)
Supplement: Supplementary file 1 [file ijms-24-08607-s001.zip › Supplementary Table S3.pdf]

Supplementary Table S3. Significantly altered proteins in the bone marrow cells of mice irradiated with 0.1Gy (A), 3Gy (2B) compared to unirradiated controls and common proteins between the two doses (C). The number of PSMs is the total number of identified peptide spectra matched for the protein. PSM: peptide spectrum matches

Table 3A<br>Deregulated proteins in the bone marrow cells of mice irradiated with 0.1Gy						
**Downregulated proteins**						
**Protein name**	**Gene Symbol**	**UniProt ID**	**PSMs**	**Unique Peptides**	**Abundance Ratio Adj. P-Value: 0.1Gy vs 0Gy**	**Abundance Ratio Adj. P-Value: 0.1Gy vs 0Gy**
RUN and FYVE domain-containing protein 1	Rufy1	Q8BIJ7	5	2	0 .01	2.23E-16
Sphingolipid delta(4)-desaturase DES1	Degs1	O09005	5	2	0 .01	2.23E-16
Exocyst complex component 4	Exoc4	O35382	8	2	0 .01	2.23E-16
Retinoblastoma-associated protein	Rb1	P13405	3	2	0 .01	2.23E-16
Exosome complex exonuclease RRP44	Dis3	Q9CSH3	3	2	0 .01	2.23E-16
Cleavage and polyadenylation specificity factor subunit 3	Cpsf3	Q9QXK7	2	2	0 .01	2.23E-16
Transmembrane and coiled-coil domains protein 1	Tmcc1	Q69ZZ6	20	2	0 .01	2.23E-16
Ankyrin repeat domain-containing protein 22	Ankrd22	Q9D3J5	9	3	0 .01	2.23E-16
Beta-galactosidase	Glb1	P23780	4	2	0 .01	2.23E-16
Peptidyl-prolyl cis-trans isomerase-like 4	Ppil4	Q9CXG3	9	4	0 .01	2.23E-16
Vacuolar protein sorting-associated protein 16 homolog	Vps16	Q920Q4	2	2	0 .01	2.23E-16
ERO1-like protein beta	Ero1b	Q8R2E9	32	2	0 .01	2.23E-16
Uncharacterized protein KIAA0513	Kiaa0513	Q8R0A7	5	2	0 .01	2.23E-16
Fatty acid-binding protein. adipocyte	Fabp4	P04117	38	5	0 .177	1.12E-07
2'-5'-oligoadenylate synthase 3	Oas3	Q8VI93	22	10	0 .208	1.35E-05
Formyl peptide receptor 2	Fpr2	O88536	31	2	0 .232	5.20E-04
Regulator of G-protein signaling 19	Rgs19	Q9CX84	11	2	0 .251	2.21E-02

G-protein coupled receptor 84	Gpr84	Q8CIM5	21	2	0 .287	4.34E-03
High affinity immunoglobulin epsilon receptor subunit gamma	Fcer1g	P20491	20	2	0 .289	1.95E-04
Cytoplasmic tyrosine-protein kinase BMX	Bmx	P97504	26	2	0 .3	2.03E-02
Protein jagunal homolog 1	Jagn1	Q5XKN4	13	2	0 .318	2.91E-02
Leukosialin	Spn	P15702	63	4	0 .329	1.39E-03
2'-5'-oligoadenylate synthase 1A	Oas1a	P11928	6	2	0 .334	4.93E-02
Leucine-rich repeat and calponin homology domain-containing protein 4	Lrch4	Q921G6	63	5	0 .335	1.81E-03
Paired immunoglobulin-like type 2 receptor alpha	Pilra	Q2YFS3	11	4	0 .371	5.15E-03
Microsomal glutathione S-transferase 1	Mgst1	Q91VS7	74	3	0 .397	1.13E-03
Cullin-5	Cul5	Q9D5V5	8	3	0 .426	4.60E-02
Glycogen [starch] synthase. muscle	Gys1	Q9Z1E4	139	13	0 .427	1.58E-02
Olfactomedin-4	Olfm4	Q3UZZ4	462	14	0 .446	1.67E-02
Annexin A2	Anxa2	P07356	1543	21	0 .475	3.47E-02
CKLF-like MARVEL transmembrane domain-containing protein 7	Cmtm7	Q9ESD6	48	3	0 .479	4.21E-02
Cytochrome b-245 heavy chain	Cybb	Q61093	1306	21	0 .479	3.75E-02
Ras-related protein Rab-32	Rab32	Q9CZE3	137	9	0 .523	4.99E-02

Upregulated proteins

Protein name	Gene Symbol	UniProt ID	PSMs	Unique Peptides	Abundance Ratio 0.1Gy vs 0Gy	Abundance Ratio Adj. P-Value: 0.1Gy vs 0Gy
Carbonic anhydrase 1	Ca1	P13634	2432	14	9 .702	2.23E-16
RNA-binding protein EWS	Ewsr1	Q61545	62	3	10 .141	2.23E-16
Hemoglobin subunit alpha	Hba	P01942	49649	17	10 .653	2.23E-16
Cathepsin E	Ctse	P70269	156	3	10 .898	2.23E-16
Glycogen synthase kinase-3 alpha	Gsk3a	Q2NL51	25	2	14 .15	2.23E-16
Hemoglobin subunit beta-2	Hbb-b2	P02089	40357	11	19 .161	2.23E-16
Latexin	Lxn	P70202	10	2	20 .084	2.23E-16
N-acylneuraminate-9-phosphatase	Nanp	Q9CPT3	8	2	100	2.23E-16

FAS-associated factor 2	Faf2	Q3TDN2	5	3	100	2.23E-16
Cleavage and polyadenylation specificity factor subunit 2	Cpsf2	O35218	12	2	100	2.23E-16
Plasminogen	Plg	P20918	8	2	100	2.23E-16
Golgi SNAP receptor complex member 2	Gosr2	O35166	6	2	100	2.23E-16
Mitochondrial import inner membrane translocase subunit Tim8 A	Timm8a 1	Q9WVA 2	9	2	100	2.23E-16
PEST proteolytic signal-containing nuclear protein	Pcnp	Q6P8I4	12	3	100	2.23E-16
Coiled-coil domain-containing protein 124	Ccdc124	Q9D8X2	4	2	100	2.23E-16
Sorting nexin-29	Snx29	Q9D3S3	2	2	100	2.23E-16
ATP-binding cassette sub-family D member 3	Abcd3	P55096	6	3	100	2.23E-16
DnaJ homolog subfamily B member 6	Dnajb6	O54946	4	2	100	2.23E-16
CD180 antigen	Cd180	Q62192	6	3	100	2.23E-16
Heat shock 70 kDa protein 14	Hspa14	Q99M31	9	6	100	2.23E-16
N-acetylglucosamine-1-phosphodiester alpha-N-acetylglucosaminidase	Nagpa	Q8BJ48	3	2	100	2.23E-16
Acyl carrier protein. mitochondrial	Ndufab1	Q9CR21	8	2	100	2.23E-16
Mitochondrial import inner membrane translocase subunit Tim13	Timm13	P62075	37	2	100	2.23E-16
Peroxiredoxin-2	Prdx2	Q61171	1685	12	8 .753	4.93E-15
Heat shock-related 70 kDa protein 2	Hspa2	P17156	1793	5	6 .769	3.12E-13
SCY1-like protein 2	Scyl2	Q8CFE4	6	2	5 .911	5.42E-12
Carbonic anhydrase 2	Ca2	P00920	4434	16	6 .377	3.87E-11
Cysteine desulfurase. mitochondrial	Nfs1	Q9Z1J3	5	2	6 .915	4.86E-11
Intron-binding protein aquarius	Aqr	Q8CFQ3	12	3	8 .603	1.41E-10
ATP synthase subunit delta. mitochondrial	Atp5d	Q9D3D9	74	4	7 .285	7.17E-10
Hemoglobin subunit beta-1	Hbb-b1	P02088	47412	14	5 .592	1.26E-09
40S ribosomal protein S15	Rps15	P62843	95	3	6 .917	1.08E-08
Alpha-hemoglobin-stabilizing protein	Ahsp	Q9CY02	318	6	4 .506	2.28E-07

WAS/WASL-interacting protein family member 1	Wipf1	Q8K1I7	36	7	5 .884	2.66E-07
Hydroxyacylglutathione hydrolase. mitochondrial	Hagh	Q99KB8	109	7	4 .843	3.43E-07
V-type proton ATPase subunit G 1	Atp6v1g 1	Q9CR51	58	3	5 .208	3.49E-07
Calcium-regulated heat stable protein 1	Carhsp1	Q9CR86	34	3	5 .495	3.55E-07
10 kDa heat shock protein. mitochondrial	Hspe1	Q64433	238	4	4 .092	4.35E-07
Serum albumin	9913 GN	P02769	10906	40	4 .139	1.40E-06
Trans-2-enoyl-CoA reductase. mitochondrial	Mecr	Q9DCS3	3	2	8 .049	3.53E-06
RNA-binding protein FUS	Fus	P56959	43	4	5 .333	4.45E-06
Oxygen-dependent coproporphyrinogen-III oxidase. mitochondrial	Cpox	P36552	559	18	3 .873	5.45E-06
Prothymosin alpha	Ptma	P26350	331	7	3 .759	9.84E-06
E3 ubiquitin-protein ligase BRE1B	Rnf40	Q3U319	13	2	4 .783	1.52E-05
Alpha-2-HS-glycoprotein	Ahsg	P29699	153	2	3 .636	1.87E-05
Vesicle-associated membrane protein-associated protein A	Vapa	Q9WV5 5	237	2	4 .602	2.85E-05
Superoxide dismutase [Cu-Zn]	Sod1	P08228	207	5	3 .284	3.42E-05
Glutathione S-transferase Mu 5	Gstm5	P48774	217	4	4 .319	4.20E-05
Porphobilinogen deaminase	Hmbs	P22907	897	18	3 .357	8.25E-05
Stathmin	Stmn1	P54227	190	6	3 .35	8.58E-05
Histidine triad nucleotide-binding protein 1	Hint1	P70349	83	3	3 .494	1.09E-04
Ankyrin-3	Ank3	G5E8K5	71	2	4 .137	1.13E-04
Baculoviral IAP repeat-containing protein 6	Birc6	O88738	12	2	4 .473	2.67E-04
Heterogeneous nuclear ribonucleoprotein D-like	Hnrnpdl	Q9Z130	299	3	3 .249	3.93E-04
Cytochrome c oxidase subunit 5B. mitochondrial	Cox5b	P19536	76	3	3 .741	4.30E-04
ATP-binding cassette sub-family B member 6. mitochondrial	Abcb6	Q9DC29	11	2	5 .523	5.06E-04
Methionine aminopeptidase 2	Metap2	O08663	299	14	2 .766	5.99E-04
Putative transferase CAF17 homolog. mitochondrial	Iba57	Q8CAK1	21	3	3.13	5.99E-04

Spectrin alpha chain. erythrocytic 1	Spta1	P08032	2673	101	2 .973	6.48E-04
Ganglioside GM2 activator	Gm2a	Q60648	71	3	3 .287	1.27E-03
Ankyrin-1	Ank1	Q02357	1771	65	2 .82	1.49E-03
Nuclear pore glycoprotein p62	Nup62	Q63850	12	2	4 .56	1.54E-03
Galectin-1	Lgals1	P16045	227	8	2 .572	1.61E-03
Band 3 anion transport protein	Slc4a1	P04919	3120	27	2 .781	1.81E-03
IgG receptor FcRn large subunit p51	Fcgrt	Q61559	13	2	4 .721	1.81E-03
Cytochrome b-c1 complex subunit 6. mitochondrial	Uqcrh	P99028	73	2	3 .512	2.11E-03
40S ribosomal protein S12	Rps12	P63323	421	6	2 .709	2.66E-03
Catalase	Cat	P24270	1187	25	2 .682	3.09E-03
Endonuclease domain-containing 1 protein	Endod1	Q8C522	23	4	3 .253	3.18E-03
Frataxin. mitochondrial	Fxn	O35943	31	3	3 .142	3.53E-03
Ig kappa chain C region	1 SV	P01837	99	4	3 .037	3.61E-03
60S acidic ribosomal protein P1	Rplp1	P47955	165	3	2 .501	4.24E-03
Erythrocyte membrane protein band 4.2	Epb42	P49222	396	21	2 .369	4.55E-03
Microtubule-associated protein 4	Map4	P27546	9	5	5 .411	5.35E-03
Metalloproteinase inhibitor 3	Timp3	P39876	7	2	4 .015	6.42E-03
SH3 domain-binding glutamic acid-rich-like protein	Sh3bgrl	Q9JJU8	165	5	3 .002	6.56E-03
Zyxin	Zyx	Q62523	116	10	2 .917	6.90E-03
Hematopoietic lineage cell-specific protein	Hcls1	P49710	71	9	2 .825	8.02E-03
ATP synthase subunit d. mitochondrial	Atp5h	Q9DCX2	103	7	2 .919	9.22E-03
39S ribosomal protein L12. mitochondrial	Mrpl12	Q9DB15	49	4	2 .893	9.23E-03
Cytosolic 5'-nucleotidase 3A	Nt5c3a	Q9D020	94	10	2 .941	1.11E-02
Tripeptidyl-peptidase 1	Tpp1	O89023	32	3	2 .822	1.17E-02
Inter-alpha-trypsin inhibitor heavy chain H2	Itih2	Q61703	17	4	3 .417	1.20E-02
Stefin-2	Stfa2	P35174	42	2	3 .665	1.26E-02
Elongation factor 1-delta	Eef1d	P57776	464	9	2 .439	1.28E-02
Serpin H1	Serpinh1	P19324	22	4	3 .683	1.28E-02
Elongation factor 1-beta	Eef1b	O70251	354	7	2 .305	1.34E-02
Stefin-1	Stfa1	P35175	207	4	2 .434	1.51E-02

Cytochrome c oxidase subunit 6B1	Cox6b1	P56391	215	5	2 .439	1.80E-02
Asparagine synthetase [glutamine-hydrolyzing]	Asns	Q61024	41	5	2 .71	2.03E-02
Mitochondrial 2-oxodicarboxylate carrier	Slc25a21	Q8BZ09	15	3	3 .466	2.10E-02
Thioredoxin domain-containing protein 5	Txndc5	Q91W90	203	11	2 .254	2.10E-02
Protein deglycase DJ-1	Park7	Q99LX0	228	8	2 .34	2.42E-02
Platelet glycoprotein 4	Cd36	Q08857	104	7	2 .393	2.43E-02
Transcription factor PU.1	Spi1	P17433	41	4	2 .529	2.49E-02
Redox-regulatory protein FAM213A	Fam213a	Q9CYH2	8	3	3 .371	2.49E-02
5'(3')-deoxyribonucleotidase. cytosolic type	Nt5c	Q9JM14	102	6	2 .524	2.85E-02
DnaJ homolog subfamily A member 4	Dnaja4	Q9JMC3	2	2	3 .847	2.92E-02
Heme-binding protein 1	Hebp1	Q9R257	252	6	2 .393	2.97E-02
Carbonyl reductase [NADPH] 1	Cbr1	P48758	17	5	2 .514	3.14E-02
Peroxiredoxin-4	Prdx4	O08807	230	6	2 .356	3.46E-02
Acetyl-CoA acetyltransferase. mitochondrial	Acat1	Q8QZT1	53	6	2 .519	3.46E-02
Keratin. type II cuticular Hb4	Krt84	Q99M73	391	2	3 .344	3.52E-02
Trem-like transcript 1 protein	Treml1	Q8K558	72	4	2 .55	3.72E-02
Interleukin-36 gamma	Il36g	Q8R460	49	4	2 .837	3.82E-02
Partner of Y14 and mago	Pym1	Q8CHP5	35	3	2 .482	4.45E-02
ADP-sugar pyrophosphatase	Nudt5	Q9JKX6	13	3	3 .762	4.46E-02
Prefoldin subunit 6	Pfdn6	Q03958	30	2	2 .525	4.59E-02
Hepatocyte growth factor-regulated tyrosine kinase substrate	Hgs	Q99LI8	19	2	2 .417	4.93E-02

Table 3B
Deregulated proteins in the bone marrow cells of mice irradiated with 3Gy

Downregulated proteins

**Protein name**	**Gene Symbol**	**UniProt ID**	**PSMs**	**Unique Peptides**	**Abundance Ratio 3Gy vs 0Gy**	**Abundance Ratio Adj. P-Value: 3Gy vs 0Gy**
Carbonyl reductase [NADPH] 2	Cbr2	P08074	2	2	0 .01	6.12E-17

RUN and FYVE domain-containing protein 1	Rufy1	Q8BIJ7	5	2	0 .01	6.12E-17
Sphingolipid delta(4)-desaturase DES1	Degs1	O09005	5	2	0 .01	6.12E-17
Exocyst complex component 4	Exoc4	O35382	8	2	0 .01	6.12E-17
RNA-binding protein FUS	Fus	P56959	43	4	0 .01	6.12E-17
40S ribosomal protein S28	Rps28	P62858	103	2	0 .01	6.12E-17
Dynein light chain roadblock-type 1	Dynlrb1	P62627	20	3	0 .01	6.12E-17
Beta-2-microglobulin	B2m	P01887	31	2	0 .01	6.12E-17
39S ribosomal protein L12. mitochondrial	Mrpl12	Q9DB15	49	4	0 .01	6.12E-17
Nuclear pore glycoprotein p62	Nup62	Q63850	12	2	0 .01	6.12E-17
F-box only protein 50	Nccrp1	G3X9C2	6	3	0 .01	6.12E-17
Metalloproteinase inhibitor 3	Timp3	P39876	7	2	0 .01	6.12E-17
Thioredoxin. mitochondrial	Txn2	P97493	6	2	0 .01	6.12E-17
Vacuolar protein sorting-associated protein 18 homolog	Vps18	Q8R307	7	2	0 .01	6.12E-17
Casein kinase II subunit beta	Csnk2b	P67871	68	3	0 .01	6.12E-17
Muscleblind-like protein 2	Mbnl2	Q8C181	9	3	0 .01	6.12E-17
Galectin-related protein	Lgalsl	Q8VED9	3	2	0 .01	6.12E-17
Protein canopy homolog 3	Cnpy3	Q9DAU1	33	4	0 .01	6.12E-17
Granulins	Grn	P28798	11	2	0 .01	6.12E-17
CGG triplet repeat-binding protein 1	Cggbp1	Q8BHG9	5	2	0 .01	6.12E-17

NADH dehydrogenase [ubiquinone] 1 beta subcomplex subunit 9	Ndufb9	Q9CQJ8	19	2	0 .01	6.12E-17
Poly(A)-specific ribonuclease PARN	Parn	Q8VDG 3	3	2	0 .01	6.12E-17
C-Myc-binding protein	Mycbp	Q9EQS3	26	3	0 .01	6.12E-17
Ragulator complex protein LAMTOR1	Lamtor1	Q9CQ22	23	3	0 .01	6.12E-17
Tumor protein D52	Tpd52	Q62393	19	3	0 .01	6.12E-17
Myosin light chain 4	Myl4	P09541	8	4	0 .01	6.12E-17
Phosphoacetylglucosamine mutase	Pgm3	Q9CYR6	2	2	0 .01	6.12E-17
ATP synthase-coupling factor 6. mitochondrial	Atp5j	P97450	33	4	0 .01	6.12E-17
Dedicator of cytokinesis protein 10	Dock10	Q8BZN6	14	3	0 .01	6.12E-17
Ubiquitin-associated protein 2-like	Ubap2l	Q80X50	7	2	0 .01	6.12E-17
Nuclear pore complex protein Nup50	Nup50	Q9JIH2	32	4	0 .01	6.12E-17
Serine/threonine-protein kinase TAO3	Taok3	Q8BYC6	8	2	0 .01	6.12E-17
Transmembrane protein 165	Tmem16 5	P52875	18	2	0 .01	6.12E-17
U6 snRNA-associated Sm-like protein LSm5	Lsm5; LOC100 044729; LOC102 641341	P62322	6	2	0 .01	6.12E-17
Elongation of very long chain fatty acids protein 1	Elovl1	Q9JLJ5	11	2	0 .01	6.12E-17
Exosome complex component RRP45	Exosc9	Q9JHI7	7	3	0 .01	6.12E-17

Peptidylprolyl isomerase domain and WD repeat-containing protein 1	Ppwd1	Q8CEC6	10	4	0 .01	6.12E-17
Transcription initiation factor IIB	Gtf2b	P62915	22	2	0 .01	6.12E-17
Serine/threonine-protein kinase 24	Stk24	Q99KH8	90	2	0 .01	6.12E-17
Histone H3-like centromeric protein A	Cenpa	O35216	11	2	0 .01	6.12E-17
DNA primase small subunit	Prim1	P20664	35	3	0 .01	6.12E-17
Cleavage stimulation factor subunit 2	Cstf2	Q8BIQ5	23	3	0 .01	6.12E-17
Transmembrane protein 214	Tmem21 4	Q8BM55	4	2	0 .01	6.12E-17
TRAF3-interacting JNK-activating modulator	Traf3ip3	Q8C0G2	14	3	0 .01	6.12E-17
H-2 class II histocompatibility antigen. I-A beta chain	H2-Eb1	P18468	61	3	0 .01	6.12E-17
U4/U6 small nuclear ribonucleoprotein Prp3	Prpf3	Q922U1	13	3	0 .01	6.12E-17
Microtubule-associated protein 4	Map4; Mtap4	P27546	9	5	0 .01	6.12E-17
Exosome complex exonuclease RRP44	Dis3	Q9CSH3	3	2	0 .01	6.12E-17
Integrator complex subunit 7	Ints7	Q7TQK1	5	3	0 .01	6.12E-17
Dual specificity mitogen-activated protein kinase kinase 2	Map2k2	Q63932	21	2	0 .01	6.12E-17
Serine/arginine repetitive matrix protein 1	Srrm1	Q52KI8	8	2	0 .01	6.12E-17

WD repeat-containing protein 82	Wdr82	Q8BFQ4	17	3	0 .01	6.12E-17
NADH-ubiquinone oxidoreductase chain 4	ND4	P03911	26	2	0 .01	6.12E-17
Cleavage and polyadenylation specificity factor subunit 3	Cpsf3	Q9QXK 7	2	2	0 .01	6.12E-17
C-terminal-binding protein 2	Ctbp2	P56546	69	2	0 .01	6.12E-17
Transmembrane and coiled-coil domains protein 1	Tmcc1	Q69ZZ6	20	2	0 .01	6.12E-17
Regulator of G-protein signaling 14	Rgs14	P97492	4	2	0 .01	6.12E-17
BolA-like protein 1	Bola1	Q9D8S9	3	2	0 .01	6.12E-17
DNA polymerase delta subunit 2	Pold2	O35654	5	2	0 .01	6.12E-17
Syntaxin-5	Stx5a	Q8K1E0	9	3	0 .01	6.12E-17
Golgin subfamily A member 7	Golga7	Q91W53	4	2	0 .01	6.12E-17
Mitochondrial import receptor subunit TOM34	Tomm34	Q9CYG7	31	3	0 .01	6.12E-17
Pyruvate dehydrogenase protein X component. mitochondrial	Pdhx	Q8BKZ9	3	2	0 .01	6.12E-17
RNA-binding protein 12	Rbm12	Q8R4X3	3	2	0 .01	6.12E-17
Exosome complex exonuclease RRP42	Exosc7	Q9D0M0	13	4	0 .01	6.12E-17
N-alpha-acetyltransferase 25. NatB auxiliary subunit	Naa25	Q8BWZ 3	14	3	0 .01	6.12E-17
Mitochondrial 2-oxodicarboxylate carrier	Slc25a21	Q8BZ09	15	3	0 .01	6.12E-17

Serine/threonine-protein kinase Nek9	Nek9	Q8K1R7	18	4	0 .01	6.12E-17
Ras association domain-containing protein 2	Rassf2	Q8BMS9	16	4	0 .01	6.12E-17
NADH dehydrogenase [ubiquinone] 1 alpha subcomplex subunit 11	Ndufa11	Q9D8B4	14	3	0 .01	6.12E-17
Histone deacetylase 2	Hdac2	P70288	80	3	0 .01	6.12E-17
Bifunctional methylenetetrahydrofolate dehydrogenase/cyclohydrolase. mitochondrial	Mthfd2	P18155	7	2	0 .01	6.12E-17
Ras GTPase-activating protein-binding protein 2	G3bp2	P97379	26	3	0 .01	6.12E-17
Condensin-2 complex subunit G2	Ncapg2	Q6DFV1	8	3	0 .01	6.12E-17
Nesprin-3	4831426I19Rik; Syne3	Q4FZC9	8	2	0 .01	6.12E-17
Beta-galactosidase	Glb1	P23780	4	2	0 .01	6.12E-17
Unconventional myosin-Ic	Myo1c	Q9WTI7	25	3	0 .01	6.12E-17
Eukaryotic translation initiation factor 2 subunit 3. Y-linked	Eif2s3y	Q9Z0N2	243	2	0 .01	6.12E-17
Cleavage stimulation factor subunit 3	Cstf3	Q99LI7	5	3	0 .01	6.12E-17
Mitochondrial import inner membrane translocase subunit Tim9	Timm9	Q9WV98	15	2	0 .01	6.12E-17

Nucleolysin TIA-1	Tia1	P52912	14	2	0 .01	6.12E-17
THO complex subunit 1	Thoc1	Q8R3N6	10	3	0 .01	6.12E-17
Oxysterol-binding protein-related protein 9	Osbpl9	A2A8Z1	8	2	0 .01	6.12E-17
Exosome complex component RRP41	Exosc4	Q921I9	10	2	0 .01	6.12E-17
Alpha-taxilin	Txlna	Q6PAM 1	6	3	0 .01	6.12E-17
Sperm-associated antigen 7	Spag7	Q7TNE3	5	2	0 .01	6.12E-17
Protein Hook homolog 3	Hook3	Q8BUK6	26	4	0 .01	6.12E-17
Replication factor C subunit 5	Rfc5	Q9D0F6	8	3	0 .01	6.12E-17
Leucine-rich repeat protein SHOC-2	Shoc2	O88520	17	2	0 .01	6.12E-17
Polycomb protein EED	Eed	Q921E6	16	3	0 .01	6.12E-17
Kinesin-like protein KIF11	Kif11	Q6P9P6	3	3	0 .01	6.12E-17
Polypeptide N-acetylgalactosaminyltransf erase 2	Galnt2	Q6PB93	10	2	0 .01	6.12E-17
Integrator complex subunit 3	Ints3	Q7TPD0	20	5	0 .01	6.12E-17
Leucine-rich repeat serine/threonine-protein kinase 2	Lrrk2	Q5S006	3	3	0 .01	6.12E-17
RUS1 family protein C16orf58 homolog	BC01715 8	Q91W34	10	2	0 .01	6.12E-17
Mitochondrial dicarboxylate carrier	Slc25a10 ; 0610009 L18Rik	Q9QZD8	12	2	0 .01	6.12E-17
SH2 domain-containing protein 3C	Sh2d3c	Q9QZS8	7	2	0 .01	6.12E-17
Protein ABHD16A	Abhd16a	Q9Z1Q2	5	2	0 .01	6.12E-17

Active breakpoint cluster region-related protein	Abr	Q5SSL4	11	2	0 .01	6.12E-17
N-acetylneuraminate lyase	Npl	Q9DCJ9	2	2	0 .01	6.12E-17
Interleukin-1 receptor antagonist protein	Il1rn	P25085	12	2	0 .01	6.12E-17
SAFB-like transcription modulator	Sltm	Q8CH25	12	3	0 .01	6.12E-17
Aspartate--tRNA ligase. mitochondrial	Dars2	Q8BIP0	16	2	0 .01	6.12E-17
Vacuolar protein sorting-associated protein 16 homolog	Vps16	Q920Q4	2	2	0 .01	6.12E-17
Coiled-coil domain-containing protein 6	Ccdc6	D3YZP9	4	2	0 .01	6.12E-17
Huntingtin	Htt	P42859	7	2	0 .01	6.12E-17
Flotillin-1	Flot1	O08917	54	7	0 .01	6.12E-17
3-phosphoinositide-dependent protein kinase 1	Pdpk1	Q9Z2A0	3	2	0 .01	6.12E-17
Integrator complex subunit 1	Ints1	Q6P4S8	10	2	0 .01	6.12E-17
Redox-regulatory protein FAM213A	5730469 M10Rik; Fam213a	Q9CYH2	8	3	0 .01	6.12E-17
Nuclear factor NF-kappa-B p105 subunit	Nfkb1	P25799	29	3	0 .01	6.12E-17
Argininosuccinate synthase	Ass1	P16460	10	2	0 .01	6.12E-17
Plasma membrane calcium-transporting ATPase 1	Atp2b1	G5E829	28	2	0 .01	6.12E-17
Torsin-1A	Tor1a	Q9ER39	8	3	0 .01	6.12E-17

Ribosomal RNA processing protein 1 homolog A	Rrp1	P56183	15	3	0 .01	6.12E-17
Protein FAM98A	Fam98a	Q3TJZ6	4	3	0 .01	6.12E-17
Formin-like protein 3	Fmnl3	Q6ZPF4	3	2	0 .01	6.12E-17
Gamma-soluble NSF attachment protein	Napg	Q9CWZ 7	3	2	0 .01	6.12E-17
Bromodomain adjacent to zinc finger domain protein 1A	Baz1a	O88379	29	5	0 .01	6.12E-17
E3 ubiquitin-protein ligase TRIM56	Trim56	Q80VI1	3	2	0 .01	6.12E-17
Plexin-B2	Plxnb2	B2RXS4	12	3	0 .01	6.12E-17
Protein virilizer homolog	1110037 F02Rik; Virma	A2AIV2	5	3	0 .01	6.12E-17
Interferon-induced transmembrane protein 2	Ifitm2	Q99J93	2	2	0 .01	6.12E-17
Nuclear pore complex protein Nup88	Nup88	Q8CEC0	15	3	0 .01	6.12E-17
COMM domain-containing protein 7	Commd7	Q8BG94	11	2	0 .01	6.12E-17
Cytochrome c oxidase subunit 1	COX1	P00397	32	2	0 .01	6.12E-17
Sodium- and chloride-dependent glycine transporter 1	Slc6a9	P28571	2	2	0 .01	6.12E-17
Nuclear transcription factor Y subunit gamma	Nfyc	P70353	30	3	0 .01	6.12E-17
RNA polymerase II subunit A C-terminal domain phosphatase SSU72	Ssu72	Q9CY97	5	2	0 .01	6.12E-17

DNA-directed RNA polymerase II subunit RPB7	Polr2g	P62488	10	3	0 .01	6.12E-17
VIP36-like protein	Lman2l	P59481	4	3	0 .01	6.12E-17
Solute carrier family 12 member 6	Slc12a6	Q924N4	6	2	0 .01	6.12E-17
SWI/SNF-related matrix-associated actin-dependent regulator of chromatin subfamily B member 1	Smarcb1	Q9Z0H3	13	3	0 .01	6.12E-17
MOB kinase activator 3A	Mob3a	Q8BSU7	19	2	0 .01	6.12E-17
Dihydropyrimidinase-related protein 2	Dpysl2	O08553	32	5	0 .01	6.12E-17
Vacuolar protein sorting-associated protein 13C	Vps13c	Q8BX70	3	2	0 .01	6.12E-17
Myotubularin-related protein 3	Mtmr3	Q8K296	15	3	0 .01	6.12E-17
5'-nucleotidase domain-containing protein 1	Nt5dc1	Q8C5P5	28	4	0 .01	6.12E-17
Coiled-coil and C2 domain-containing protein 1B	Cc2d1b	Q8BRN9	5	2	0 .01	6.12E-17
Cleavage stimulation factor subunit 1	Cstf1	Q99LC2	5	2	0 .01	6.12E-17
Cytoplasmic FMR1-interacting protein 1	Cyfip1	Q7TMB 8	184	4	0 .01	6.12E-17
Peptidase inhibitor 16	Pi16	Q9ET66	13	2	0 .01	6.12E-17
Rab11 family-interacting protein 1	Rab11fip 1	Q9D620	24	3	0 .01	6.12E-17
Ufm1-specific protease 2	Ufsp2	Q99K23	11	2	0 .01	6.12E-17

E3 ubiquitin-protein ligase Itchy	Itch	Q8C863	3	2	0 .01	6.12E-17
Exosome complex component RRP4	Exosc2	Q8VBV3	6	3	0 .01	6.12E-17
Septin-5	Sept5	Q9Z2Q6	60	6	0 .01	6.12E-17
Transmembrane 9 superfamily member 4	Tm9sf4	Q8BH24	38	3	0 .01	6.12E-17
Microsomal glutathione S-transferase 3	Mgst3	Q9CPU4	24	2	0 .01	6.12E-17
Synaptotagmin-like protein 1	Sytl1	Q99N80	3	2	0 .01	6.12E-17
Phosphatidylinositol 3.4.5-trisphosphate-dependent Rac exchanger 1 protein	Prex1	Q69ZK0	20	5	0 .01	6.12E-17
Glycosylphosphatidylinositol anchor attachment 1 protein	Gpaa1	Q9WTK3	25	3	0 .01	6.12E-17
THUMP domain-containing protein 1	Thumpd1	Q99J36	7	2	0 .01	6.12E-17
DNA mismatch repair protein Msh6	Msh6	P54276	23	3	0 .01	6.12E-17
Dihydroxyacetone phosphate acyltransferase	Gnpat	P98192	11	3	0 .01	6.12E-17
Ribonuclease T2	Rnaset2a; Rnaset2b	Q9CQ01	7	2	0 .01	6.12E-17
DCC-interacting protein 13-alpha	Appl1	Q8K3H0	10	2	0 .01	6.12E-17
D-tyrosyl-tRNA(Tyr) deacylase 1	Dtd1	Q9DD18	12	2	0 .01	6.12E-17
WASH complex subunit SWIP	A230046K03Rik; Washc4	Q3UMB9	4	2	0 .01	6.12E-17

Friend leukemia integration 1 transcription factor	Fli1	P26323	8	4	0 .01	6.12E-17
Ribosomal L1 domain-containing protein 1	Rsl1d1	Q8BVY0	18	5	0 .01	6.12E-17
Diacylglycerol kinase zeta	Dgkz	Q80UP3	9	2	0 .01	6.12E-17
RNA-binding protein 27	Rbm27	Q5SFM8	15	2	0 .01	6.12E-17
Mitochondrial import receptor subunit TOM40 homolog	Tomm40	Q9QYA 2	30	4	0 .01	6.12E-17
Dolichol-phosphate mannosyltransferase subunit 1	Dpm1	O70152	19	5	0 .01	6.12E-17
28S ribosomal protein S26. mitochondrial	Mrps26	Q80ZS3	5	3	0 .01	6.12E-17
Hepatoma-derived growth factor-related protein 2	Hdgfrp2; Hdgfl2	Q3UMU 9	51	3	0 .01	6.12E-17
DNA polymerase epsilon subunit 3	Pole3	Q9JKP7	27	2	0 .01	6.12E-17
Propionyl-CoA carboxylase alpha chain. mitochondrial	Pcca	Q91ZA3	7	3	0 .01	6.12E-17
Enhancer of mRNA-decapping protein 4	Edc4	Q3UJB9	4	2	0 .01	6.12E-17
Dynactin subunit 4	Dctn4	Q8CBY8	12	2	0 .01	6.12E-17
Alpha-1.3-mannosyl-glycoprotein 2-beta-N-acetylglucosaminyltransfer ase	Mgat1	P27808	3	3	0 .01	6.12E-17

Mitochondrial import inner membrane translocase subunit TIM16	Pam16	Q9CQV1	34	3	0 .01	6.12E-17
Interferon-induced 35 kDa protein homolog	Ifi35	Q9D8C4	3	2	0 .01	6.12E-17
ERO1-like protein beta	Ero1lb	Q8R2E9	32	2	0 .01	6.12E-17
2'-5'-oligoadenylate synthase 1A	Oas1a	P11928	6	2	0 .01	6.12E-17
Coiled-coil domain-containing protein 90B. mitochondrial	Ccdc90b	Q8C3X2	4	2	0 .01	6.12E-17
Acetolactate synthase-like protein	Ilvbl	Q8BU33	8	2	0 .01	6.12E-17
Wings apart-like protein homolog	Wapal; Wapl	Q65Z40	3	2	0 .01	6.12E-17
Estradiol 17-beta-dehydrogenase 8	H2-Ke6	P50171	13	3	0 .01	6.12E-17
CCR4-NOT transcription complex subunit 7	Cnot7	Q60809	15	4	0 .01	6.12E-17
CD166 antigen	Alcam	Q61490	14	5	0 .01	6.12E-17
Cysteine-rich with EGF-like domain protein 2	Creld2	Q9CYA0	19	3	0 .01	6.12E-17
WASH complex subunit strumpellin	E430025 E21Rik; Washc5	Q8C2E7	12	2	0 .01	6.12E-17
Mitogen-activated protein kinase kinase kinase MLT	B230120 H23Rik; Zak; Map3k20	Q9ESL4	4	2	0 .01	6.12E-17
Peroxisomal membrane protein PEX14	Pex14	Q9R0A0	17	2	0 .01	6.12E-17

Inositol 1.4.5-trisphosphate receptor type 2	Itpr2	Q9Z329	15	3	0 .01	6.12E-17
Nucleolar transcription factor 1	Ubtf	P25976	47	6	0 .01	6.12E-17
General transcription factor II-I	Gtf2i	Q9ESZ8	13	3	0 .01	6.12E-17
Dynamin-like 120 kDa protein. mitochondrial	Opa1	P58281	21	5	0 .01	6.12E-17
Erlin-1	Erlin1	Q91X78	47	3	0 .01	6.12E-17
COMM domain-containing protein 1	Commd1	Q8K4M5	4	2	0 .01	6.12E-17
E3 ubiquitin-protein ligase MARCH5	March5	Q3KNM 2	16	2	0 .01	6.12E-17
Myotubularin-related protein 9	Mtmr9	Q9Z2D0	8	2	0 .01	6.12E-17
LETM1 domain-containing protein 1	Letmd1	Q924L1	6	2	0 .01	6.12E-17
Mitochondrial Rho GTPase 1	Rhot1	Q8BG51	6	3	0 .01	6.12E-17
Elongation factor Ts. mitochondrial	Tsfm	Q9CZR8	44	5	0 .01	6.12E-17
Transducin beta-like protein 2	Tbl2	Q9R099	13	3	0 .01	6.12E-17
Integrator complex subunit 5	Ints5	Q8CHT3	6	2	0 .01	6.12E-17
p21-activated protein kinase-interacting protein 1	Pak1ip1	Q9DCE5	7	2	0 .01	6.12E-17
Vesicle-associated membrane protein-associated protein B	Vapb	Q9QY76	90	2	0 .01	6.12E-17
Rab3 GTPase-activating protein non-catalytic subunit	Rab3gap 2	Q8BMG 7	14	2	0 .01	6.12E-17

WD repeat-containing protein 48	Wdr48	Q8BH57	20	2	0 .01	6.12E-17
Protein wntless homolog	Wls	Q6DID7	11	2	0 .01	6.12E-17
Myotubularin-related protein 6	Mtmr6	Q8VE11	3	2	0 .01	6.12E-17
Glutamine--fructose-6-phosphate aminotransferase [isomerizing] 1	Gfpt1	P47856	12	5	0 .01	6.12E-17
N-myc-interactor	Nmi	O35309	6	3	0 .01	6.12E-17
Ubiquitin-like protein 4A	Ubl4; Ubl4a; Slc10a3-ubl4; Gm38419; SlcUbl4a; Gm44504	P21126	13	3	0 .01	6.12E-17
Nucleolar GTP-binding protein 1	Gtpbp4	Q99ME9	6	3	0 .01	6.12E-17
Transcriptional repressor CTCF	Ctcf	Q61164	15	3	0 .01	6.12E-17
Protein PML	Pml	Q60953	53	6	0 .01	6.12E-17
Transforming acidic coiled-coil-containing protein 1	Tacc1	Q6Y685	11	2	0 .01	6.12E-17
WD repeat-containing protein 43	Wdr43	Q6ZQL4	16	3	0 .01	6.12E-17
Caspase-7	Casp7	P97864	34	4	0 .01	6.12E-17
Nucleoside diphosphate-linked moiety X motif 19	Nudt19	P11930	8	3	0 .01	6.12E-17
Calcium uptake protein 2. mitochondrial	Efha1; Micu2	Q8CD10	26	2	0 .01	6.12E-17
Negative elongation factor D	Nelfcd; Th1l	Q922L6	16	3	0 .01	6.12E-17

Serine/threonine-protein phosphatase 4 regulatory subunit 2	Ppp4r2	Q0VGB7	2	2	0 .01	6.12E-17
Coronin-2A	Coro2a	Q8C0P5	12	2	0 .01	6.12E-17
Arf-GAP domain and FG repeat-containing protein 1	Agfg1	Q8K2K6	9	2	0 .01	6.12E-17
Ectonucleoside triphosphate diphosphohydrolase 5	Entpd5	Q9WUZ 9	39	3	0 .01	6.12E-17
Uncharacterized protein KIAA0513	6430548 M08Rik	Q8R0A7	5	2	0 .01	6.12E-17
Keratin. type II cytoskeletal 73	Krt73	Q6NXH 9	1538	2	0 .011	6.12E-17
Echinoderm microtubule-associated protein-like 3	Eml3	Q8VC03	12	2	0 .053	3.96E-04
Kinesin-like protein KIFC1	Kifc1; Kifc5b	Q9QWT 9	11	3	0 .071	1.52E-04
PC4 and SFRS1-interacting protein	Psip1	Q99JF8	88	4	0 .095	2.42E-04
Protein kish-A	Tmem16 7	Q9CR64	28	2	0 .117	1.31E-02
U6 snRNA-associated Sm-like protein LSm2	Lsm2	O35900	71	3	0 .135	4.27E-03
Serine beta-lactamase-like protein LACTB. mitochondrial	Lactb	Q9EP89	18	5	0 .17	1.70E-02
N-acetylglucosamine-6-sulfatase	Gns	Q8BFR4	11	4	0 .188	2.89E-02
Superkiller viralicidic activity 2-like 2	Skiv2l2	Q9CZU3	67	7	0 .188	3.41E-02
ATP-dependent RNA helicase DDX39A	Ddx39	Q8VDW 0	547	4	0 .209	3.23E-02
60S ribosomal protein L35	Rpl35	Q6ZWV 7	186	3	0 .235	3.18E-02

Histone H1.5	Hist1h1b	P43276	666	7	0 .238	1.04E-02
ATP-binding cassette sub-family D member 1	Abcd1	P48410	6	3	0 .283	3.34E-02
60S ribosomal protein L18	Rpl18	P35980	356	5	0 .294	3.80E-02

Upregulated proteins

Protein name	Gene Symbol	UniProt ID	PSMs	Unique Peptides	Abundance Ratio 3Gy vs 0Gy	Abundance Ratio Adj. P-Value: 3Gy vs 0Gy
E3 ubiquitin-protein ligase BRE1B	Rnf40	Q3U319	13	2	37 .576	6.12E-17
Intron-binding protein aquarius	Aqr	Q8CFQ3	12	3	50 .766	6.12E-17
Hemoglobin subunit beta-1	Hbb-b1; Beta-s; Hbb-bs; Hbb-bt	P02088	47412	14	51 .682	6.12E-17
Hemoglobin subunit beta-2	Hbb-b2	P02089	40357	11	60 .183	6.12E-17
Glycogen synthase kinase-3 alpha	Gsk3a	Q2NL51	25	2	81 .387	6.12E-17
N-acylneuraminate-9-phosphatase	Nanp	Q9CPT3	8	2	100	6.12E-17
Plasminogen	Plg	P20918	8	2	100	6.12E-17
Golgi SNAP receptor complex member 2	Gosr2	O35166	6	2	100	6.12E-17
Mitochondrial import inner membrane translocase subunit Tim8 A	Timm8a 1	Q9WVA 2	9	2	100	6.12E-17
Coiled-coil domain-containing protein 124	Ccdc124	Q9D8X2	4	2	100	6.12E-17
Sorting nexin-29	Snx29	Q9D3S3	2	2	100	6.12E-17
Heat shock 70 kDa protein 14	Hspa14	Q99M31	9	6	100	6.12E-17
Cysteine desulfurase. mitochondrial	Nfs1	Q9Z1J3	5	2	100	6.12E-17
Latexin	Lxn	P70202	10	2	100	6.12E-17

Putative transferase CAF17 homolog. mitochondrial	Iba57	Q8CAK1	21	3	100	6.12E-17
Myosin regulatory light polypeptide 9	Myl9	Q9CQ19	197	2	100	6.12E-17
Hemoglobin subunit alpha	Hba-a1; Hba-a2	P01942	49649	17	37 .956	1.36E-15
Peroxiredoxin-2	Prdx2	Q61171	1685	12	31 .959	2.70E-14
Carbonic anhydrase 1	Car1	P13634	2432	14	31 .527	3.50E-14
Baculoviral IAP repeat-containing protein 6	Birc6	O88738	12	2	43 .207	6.99E-14
Ficolin-2	Fcnb	O70497	108	6	29 .867	8.85E-14
SCY1-like protein 2	Scyl2	Q8CFE4	6	2	26 .979	5.10E-13
SRSF protein kinase 2	Srpk2	O54781	17	5	25 .272	3.94E-11
Carbonic anhydrase 2	Car2	P00920	4434	16	20 .283	5.19E-11
Vacuolar-sorting protein SNF8	Snf8	Q9CZ28	19	2	23 .544	1.51E-10
Magnesium transporter protein 1	Magt1	Q9CQY5	58	3	18 .795	2.66E-09
60S ribosomal protein L36a	Rpl36al; Rpl36a; Rpl36a-ps3	P83882	56	2	14 .085	2.22E-07
Cytosolic acyl coenzyme A thioester hydrolase	Acot7	Q91V12	59	5	12 .819	3.46E-07
Dedicator of cytokinesis protein 5	Dock5	B2RY04	24	7	9 .602	6.22E-07
Keratin. type II cuticular Hb4	Krt84	Q99M73	391	2	45 .688	9.46E-07
Band 3 anion transport protein	Slc4a1	P04919	3120	27	9 .585	1.49E-06
Properdin	Cfp	P11680	41	6	17 .426	1.67E-06
Hydroxyacylglutathione hydrolase. mitochondrial	Hagh	Q99KB8	109	7	11 .368	1.67E-06

40S ribosomal protein S15	Rps15	P62843	95	3	10 .832	1.77E-06
Pregnancy zone protein	Pzp	Q61838	25	10	10 .095	4.28E-06
Mitochondrial chaperone BCS1	Bcs1l	Q9CZP5	12	2	22 .175	4.77E-06
U4/U6 small nuclear ribonucleoprotein Prp31	Prpf31	Q8CCF0	16	3	9 .581	6.04E-06
Selenocysteine-specific elongation factor	Eefsec	Q9JHW4	4	2	12 .172	1.13E-05
F-box-like/WD repeat-containing protein TBL1XR1	Tbl1xr1	Q8BHJ5	21	3	14.09	1.16E-05
15 kDa selenoprotein	Sep15; Selenof	Q9ERR7	70	4	9.2	1.41E-05
Ganglioside GM2 activator	Gm2a	Q60648	71	3	13 .289	1.86E-05
Ankyrin-1	Ank1	Q02357	1771	65	7 .564	2.13E-05
CAP-Gly domain-containing linker protein 1	Clip1	Q922J3	70	15	7 .54	2.20E-05
Acetyl-CoA acetyltransferase. mitochondrial	Acat1	Q8QZT1	53	6	7.07	2.67E-05
Guanine nucleotide-binding protein G(I)/G(S)/G(O) subunit gamma-10	Gng10	Q9CXP8	11	2	10 .352	2.96E-05
Catalase	Cat	P24270	1187	25	7 .081	4.27E-05
Erythrocyte membrane protein band 4.2	Epb4.2; Epb42	P49222	396	21	6 .952	5.15E-05
Spectrin alpha chain. erythrocytic 1	Spna1; Spta1	P08032	2673	101	6 .906	5.51E-05
Secernin-3	Scrn3	Q3TMH 2	86	7	7 .684	5.66E-05
Heat shock-related 70 kDa protein 2	Hspa2	P17156	1793	5	7 .491	5.68E-05

Ankyrin-3	Ank3	G5E8K5	71	2	8 .046	5.97E-05
Pre-mRNA-processing factor 6	Prpf6	Q91YR7	7	2	9 .737	7.54E-05
Nuclear respiratory factor 1	Nrf1	Q9WU00	20	3	8 .909	8.39E-05
IgG receptor FcRn large subunit p51	Fcgrt	Q61559	13	2	9 .708	1.54E-04
Fibrinogen gamma chain	Fgg	Q8VCM7	146	12	7 .051	1.54E-04
Ubiquitin-like protein ISG15	Isg15	Q64339	111	4	5 .584	1.87E-04
Superoxide dismutase [Cu-Zn]	Sod1	P08228	207	5	5 .99	2.25E-04
Chromobox protein homolog 5	Cbx5	Q61686	70	5	7 .169	2.37E-04
55 kDa erythrocyte membrane protein	Mpp1	P70290	318	13	5 .658	3.83E-04
Calcium-dependent secretion activator 1	Cadps	Q80TJ1	4	2	8 .067	4.16E-04
Protein 4.1	Epb4.1; Epb41	P48193	201	20	5 .207	4.53E-04
Thymidine kinase. cytosolic	Tk1	P04184	24	3	8 .803	5.23E-04
Transcription factor PU.1	Sfpi1; Spi1	P17433	41	4	7 .643	5.86E-04
RNA-binding protein EWS	Ewsr1	Q61545	62	3	5 .131	9.22E-04
Serpin H1	Serpinh1	P19324	22	4	7 .792	1.20E-03
Bisphosphoglycerate mutase	Bpgm	P15327	643	14	4 .907	1.36E-03
Thioredoxin reductase 2. mitochondrial	Txnrd2	Q9JLT4	90	8	5 .183	1.54E-03
Spectrin beta chain. erythrocytic	Spnb1; Sptb	P15508	2528	90	4 .828	1.56E-03
Acylamino-acid-releasing enzyme	Apeh	Q8R146	326	19	4 .682	2.02E-03

H-2 class II histocompatibility antigen. E-K alpha chain	H2-Ea-ps	P04224	17	3	8 .572	2.02E-03
Flavin reductase (NADPH)	Blvrb	Q923D2	1070	11	4 .601	2.33E-03
Renin receptor	Atp6ap2	Q9CYN9	5	2	8 .494	2.47E-03
Glutathione S-transferase Mu 5	Gstm5	P48774	217	4	5 .466	2.84E-03
Histidine triad nucleotide-binding protein 1	Hint1	P70349	83	3	4 .663	3.17E-03
Fc receptor-like A	Fcrla	Q920A9	69	5	5 .418	3.25E-03
Stefin-1	Stfa1	P35175	207	4	4 .528	3.74E-03
Calcium-regulated heat stable protein 1	Carhsp1	Q9CR86	34	3	6 .879	4.42E-03
Cytosolic 5'-nucleotidase 3A	Nt5c3	Q9D020	94	10	4 .57	5.27E-03
Cathepsin E	Ctse	P70269	156	3	5 .585	5.66E-03
Ketosamine-3-kinase	Fn3krp	Q8K274	63	5	4 .363	5.74E-03
Unconventional myosin-XVIIIa	Myo18a	Q9JMH9	52	10	4 .49	6.74E-03
Keratin. type II cytoskeletal 71	Krt71	Q9R0H5	1161	2	5 .485	7.18E-03
SWI/SNF-related matrix-associated actin-dependent regulator of chromatin subfamily E member 1	Smarce1	O54941	52	6	5 .188	7.51E-03
Porphobilinogen deaminase	Hmbs	P22907	897	18	3 .951	7.63E-03
Monocyte differentiation antigen CD14	Cd14	P10810	7	2	4 .304	7.63E-03
Myeloid cell nuclear differentiation antigen-like protein	Mndal	D0QMC 3	21	3	4 .276	8.35E-03

Major vault protein	Mvp	Q9EQK5	35	8	3 .981	9.46E-03
Alpha-hemoglobin-stabilizing protein	Ahsp	Q9CY02	318	6	3 .798	1.02E-02
Low molecular weight phosphotyrosine protein phosphatase	Acp1; LOC631286; LOC102642088	Q9D358	57	4	3 .958	1.13E-02
Frataxin. mitochondrial	Fxn	O35943	31	3	4 .446	1.15E-02
Glutamate--cysteine ligase catalytic subunit	Gclc	P97494	137	13	4 .049	1.16E-02
Integrin alpha-IIb	Itga2b	Q9QUM0	657	24	3 .722	1.18E-02
SH3 domain-binding glutamic acid-rich-like protein	Sh3bgrl	Q9JJU8	165	5	4 .878	1.21E-02
Heterogeneous nuclear ribonucleoprotein A0	Hnrnpa0	Q9CX86	493	7	3 .62	1.44E-02
Serine/threonine-protein kinase Sgk3	Sgk3	Q9ERE3	8	2	3 .327	1.45E-02
Trafficking protein particle complex subunit 12	Trappc12	Q8K2L8	31	2	3 .579	1.56E-02
Prostaglandin reductase-3	Zadh2	Q8BGC4	4	2	4 .759	1.60E-02
Beta-adducin	Add2	Q9QYB8	42	6	3 .656	1.73E-02
Replication factor C subunit 4	Rfc4	Q99J62	45	7	3 .857	1.76E-02
Metaxin-1	Mtx1	P47802	56	7	3 .832	1.82E-02
Aquaporin-1	Aqp1	Q02013	304	4	3 .461	1.97E-02
Lactoylglutathione lyase	Glo1	Q9CPU0	647	11	3 .449	2.02E-02
Dehydrogenase/reductase SDR family member 11	Dhrs11	Q3U0B3	63	8	3 .406	2.19E-02
Glutathione S-transferase A4	Gsta4	P24472	28	4	3 .516	2.20E-02

Platelet glycoprotein Ib beta chain	Gp1bb	P56400	104	4	3 .377	2.32E-02
Ubiquitin-associated domain-containing protein 1	Ubac1	Q8VDI7	30	3	4 .417	2.34E-02
Carbonyl reductase [NADPH] 1	Cbr1	P48758	17	5	3 .428	2.77E-02
Hematopoietic lineage cell-specific protein	Hcls1	P49710	71	9	4 .254	2.78E-02
Tropomodulin-1	Tmod1	P49813	26	5	3 .771	2.80E-02
NADH dehydrogenase [ubiquinone] iron-sulfur protein 4. mitochondrial	Ndufs4	Q9CXZ1	33	3	4 .363	3.02E-02
Protein deglycase DJ-1	Park7	Q99LX0	228	8	3 .409	3.27E-02
Pyruvate dehydrogenase E1 component subunit beta. mitochondrial	Pdhb	Q9D051	28	5	4 .104	3.27E-02
5'(3')-deoxyribonucleotidase. cytosolic type	Nt5c	Q9JM14	102	6	4.14	3.38E-02
Lysosomal acid lipase/cholesteryl ester hydrolase	Lipa	Q9Z0M5	11	2	6 .973	3.40E-02
Prothymosin alpha	Ptma	P26350	331	7	3 .176	3.44E-02
Peptidyl-prolyl cis-trans isomerase FKBP5	Fkbp5	Q64378	47	5	3 .659	3.48E-02
Heterogeneous nuclear ribonucleoprotein D-like	Hnrnpdl; Hnrnpdl1 ; Hnrpdl	Q9Z130	299	3	3 .172	3.61E-02
Acid ceramidase	Asah1	Q9WV5 4	81	9	3 .199	3.71E-02
Cathepsin B	Ctsb	P10605	222	10	3 .304	3.80E-02

3-beta-hydroxysteroid-Delta(8).Delta(7)-isomerase	Ebp	P70245	40	3	2 .933	3.80E-02
Arachidonate 12-lipoxygenase. 12S-type	Alox12	P39655	23	4	4 .147	4.05E-02
Integrin alpha-2	Itga2	Q62469	10	3	6 .262	4.06E-02
Uroporphyrinogen decarboxylase	Urod	P70697	344	13	2 .999	4.29E-02
WD repeat and HMG-box DNA-binding protein 1	Wdhd1	P59328	34	6	3 .035	4.55E-02

Table 3C
Common deregulated proteins in the bone marrow cells of mice irradiated with 0.1Gy and 3Gy

			**Group**			
			**BM 0Gy vs BM 0.1Gy**		**BM 0Gy vs BM 3Gy**	
**Protein name**	**Gene Symbol**	**UniProt ID**	**Abundance Ratio 0.1Gy vs 0Gy**	**Abundance Ratio Adj. P-Value: 0.1 Gy vs 0 Gy**	**Abundance Ratio 3Gy vs 0Gy**	**Abundance Ratio Adj. P-Value: 3Gy vs 0Gy**
Uncharacterized protein KIAA0513	6430548 M08Rik	Q8R0A7	0.01	2.23E-16	0.01	6.12E-17
Cleavage and polyadenylation specificity factor subunit 3	Cpsf3	Q9QXK7	0.01	2.23E-16	0.01	6.12E-17
Sphingolipid delta(4)-desaturase DES1	Degs1	O09005	0.01	2.23E-16	0.01	6.12E-17
Exosome complex exonuclease RRP44	Dis3	Q9CSH3	0.01	2.23E-16	0.01	6.12E-17
ERO1-like protein beta	Ero1lb	Q8R2E9	0.01	2.23E-16	0.01	6.12E-17
Exocyst complex component 4	Exoc4	O35382	0.01	2.23E-16	0.01	6.12E-17
Beta-galactosidase	Glb1	P23780	0.01	2.23E-16	0.01	6.12E-17
**2'-5'-oligoadenylate synthase 1A**	**Oas1a**	**P11928**	**0.334**	**4.93E-02**	**0.01**	**6.12E-17**
RUN and FYVE domain-containing protein 1	Rufy1	Q8BIJ7	0.01	2.23E-16	0.01	6.12E-17
Transmembrane and coiled-coil domains protein 1	Tmcc1	Q69ZZ6	0.01	2.23E-16	0.01	6.12E-17
Vacuolar protein sorting-associated protein 16 homolog	Vps16	Q920Q4	0.01	2.23E-16	0.01	6.12E-17
Redox-regulatory protein FAM213A	5730469 M10Rik; Fam213a	Q9CYH2	3.371	2.49E-02	0.01	6.12E-17

**Acetyl-CoA acetyltransferase, mitochondrial**	**Acat1**	**Q8QZT1**	**2.519**	**3.46E-02**	**7.07**	**2.67E-05**
Alpha-hemoglobin-stabilizing protein	Ahsp	Q9CY02	4.506	2.28E-07	3.798	1.02E-02
**Ankyrin-1**	**Ank1**	**Q02357**	**2.82**	**1.49E-03**	**7.564**	**2.13E-05**
**Ankyrin-3**	**Ank3**	**G5E8K5**	**4.137**	**1.13E-04**	**8.046**	**5.97E-05**
**Intron-binding protein aquarius**	**Aqr**	**Q8CFQ3**	**8.603**	**1.41E-10**	**50.766**	**6.12E-17**
**Baculoviral IAP repeat-containing protein 6**	**Birc6**	**O88738**	**4.473**	**2.67E-04**	**43.207**	**6.99E-14**
**Carbonic anhydrase 1**	**Car1**	**P13634**	**9.702**	**2.23E-16**	**31.527**	**3.50E-14**
**Carbonic anhydrase 2**	**Car2**	**P00920**	**6.377**	**3.87E-11**	**20.283**	**5.19E-11**
Calcium-regulated heat stable protein 1	Carhsp1	Q9CR86	5.495	3.55E-07	6.879	4.42E-03
**Catalase**	**Cat**	**P24270**	**2.682**	**3.09E-03**	**7.081**	**4.27E-05**
Carbonyl reductase [NADPH] 1	Cbr1	P48758	2.514	3.14E-02	3.428	2.77E-02
Coiled-coil domain-containing protein 124	Ccdc124	Q9D8X2	100	2.23E-16	100	6.12E-17
Cathepsin E	Ctse	P70269	10.898	2.23E-16	5.585	5.66E-03
**Erythrocyte membrane protein band 4.2**	**Epb4.2; Epb42**	**P49222**	**2.369**	**4.55E-03**	**6.952**	**5.15E-05**
RNA-binding protein EWS	Ewsr1	Q61545	10.141	2.23E-16	5.131	9.22E-04
**IgG receptor FcRn large subunit p51**	**Fcgrt**	**Q61559**	**4.721**	**1.81E-03**	**9.708**	**1.54E-04**
RNA-binding protein FUS	Fus	P56959	5.333	4.45E-06	0.01	6.12E-17
Frataxin, mitochondrial	Fxn	O35943	3.142	3.53E-03	4.446	1.15E-02
**Ganglioside GM2 activator**	**Gm2a**	**Q60648**	**3.287**	**1.27E-03**	**13.289**	**1.86E-05**
Golgi SNAP receptor complex member 2	Gosr2	O35166	100	2.23E-16	100	6.12E-17
**Glycogen synthase kinase-3 alpha**	**Gsk3a**	**Q2NL51**	**14.15**	**2.23E-16**	**81.387**	**6.12E-17**
Glutathione S-transferase Mu 5	Gstm5	P48774	4.319	4.20E-05	5.466	2.84E-03
**Hydroxyacylglutathione hydrolase, mitochondrial**	**Hagh**	**Q99KB8**	**4.843**	**3.43E-07**	**11.368**	**1.67E-06**
**Hemoglobin subunit alpha**	**Hba-a1; Hba-a2**	**P01942**	**10.653**	**2.23E-16**	**37.956**	**1.36E-15**
**Hemoglobin subunit beta-1**	**Hbb-b1; Beta-s; Hbb-bs; Hbb-bt**	**P02088**	**5.592**	**1.26E-09**	**51.682**	**6.12E-17**
**Hemoglobin subunit beta-2**	**Hbb-b2**	**P02089**	**19.161**	**2.23E-16**	**60.183**	**6.12E-17**
**Hematopoietic lineage cell-specific protein**	**Hcls1**	**P49710**	**2.825**	**8.02E-03**	**4.254**	**2.78E-02**
Histidine triad nucleotide-binding protein 1	Hint1	P70349	3.494	1.09E-04	4.663	3.17E-03
Porphobilinogen deaminase	Hmbs	P22907	3.357	8.25E-05	3.951	7.63E-03
Heterogeneous nuclear ribonucleoprotein D-like	Hnrnpdl; Hnrnpdl1; Hnrpdl	Q9Z130	3.249	3.93E-04	3.172	3.61E-02
Heat shock 70 kDa protein 14	Hspa14	Q99M31	100	2.23E-16	100	6.12E-17

Heat shock-related 70 kDa protein 2	Hspa2	P17156	6.769	3.12E-13	7.491	5.68E-05
**Putative transferase CAF17 homolog, mitochondrial**	**Iba57**	**Q8CAK1**	**3.13**	**5.99E-04**	**100**	**6.12E-17**
**Keratin, type II cuticular Hb4**	**Krt84**	**Q99M73**	**3.344**	**3.52E-02**	**45.688**	**9.46E-07**
**Latexin**	**Lxn**	**P70202**	**20.084**	**2.23E-16**	**100**	**6.12E-17**
Microtubule-associated protein 4	Map4; Mtap4	P27546	5.411	5.35E-03	0.01	6.12E-17
39S ribosomal protein L12, mitochondrial	Mrpl12	Q9DB15	2.893	9.23E-03	0.01	6.12E-17
N-acylneuraminate-9-phosphatase	Nanp	Q9CPT3	100	2.23E-16	100	6.12E-17
**Cysteine desulfurase, mitochondrial**	**Nfs1**	**Q9Z1J3**	**6.915**	**4.86E-11**	**100**	**6.12E-17**
**5'(3')-deoxyribonucleotidase, cytosolic type**	**Nt5c**	**Q9JM14**	**2.524**	**2.85E-02**	**4.14**	**3.38E-02**
**Cytosolic 5'-nucleotidase 3A**	**Nt5c3**	**Q9D020**	**2.941**	**1.11E-02**	**4.57**	**5.27E-03**
Nuclear pore glycoprotein p62	Nup62	Q63850	4.56	1.54E-03	0.01	6.12E-17
Protein deglycase DJ-1	Park7	Q99LX0	2.34	2.42E-02	3.409	3.27E-02
Plasminogen	Plg	P20918	100	2.23E-16	100	6.12E-17
**Peroxiredoxin-2**	**Prdx2**	**Q61171**	**8.753**	**4.93E-15**	**31.959**	**2.70E-14**
Prothymosin alpha	Ptma	P26350	3.759	9.84E-06	3.176	3.44E-02
**E3 ubiquitin-protein ligase BRE1B**	**Rnf40**	**Q3U319**	**4.783**	**1.52E-05**	**37.576**	**6.12E-17**
**40S ribosomal protein S15**	**Rps15**	**P62843**	**6.917**	**1.08E-08**	**10.832**	**1.77E-06**
**SCY1-like protein 2**	**Scyl2**	**Q8CFE4**	**5.911**	**5.42E-12**	**26.979**	**5.10E-13**
**Serpin H1**	**Serpinh1**	**P19324**	**3.683**	**1.28E-02**	**7.792**	**1.20E-03**
**Transcription factor PU.1**	**Sfpi1; Spi1**	**P17433**	**2.529**	**2.49E-02**	**7.643**	**5.86E-04**
**SH3 domain-binding glutamic acid-rich-like protein**	**Sh3bgrl**	**Q9JJU8**	**3.002**	**6.56E-03**	**4.878**	**1.21E-02**
Mitochondrial 2-oxodicarboxylate carrier	Slc25a21	Q8BZ09	3.466	2.10E-02	0.01	6.12E-17
**Band 3 anion transport protein**	**Slc4a1**	**P04919**	**2.781**	**1.81E-03**	**9.585**	**1.49E-06**
Sorting nexin-29	Snx29	Q9D3S3	100	2.23E-16	100	6.12E-17
**Superoxide dismutase [Cu-Zn]**	**Sod1**	**P08228**	**3.284**	**3.42E-05**	**5.99**	**2.25E-04**
**Spectrin alpha chain, erythrocytic 1**	**Spna1; Spta1**	**P08032**	**2.973**	**6.48E-04**	**6.906**	**5.51E-05**
**Stefin-1**	**Stfa1**	**P35175**	**2.434**	**1.51E-02**	**4.528**	**3.74E-03**
Mitochondrial import inner membrane translocase subunit Tim8 A	Timm8a1	Q9WVA2	100	2.23E-16	100	6.12E-17
Metalloproteinase inhibitor 3	P39876	Timp3	4.015	6.42E-03	0.01	6.12E-17
