# Supplementary material for: The miRNA Content of Bone Marrow-Derived Extracellular Vesicles Contributes to Protein Pathway Alterations Involved in Ionising Radiation-Induced Bystander Responses
Source: Int J Mol Sci. 2023 May 11;24(10):8607. doi: 10.3390/ijms24108607 (PMC10218377; doi:10.3390/ijms24108607)
Supplement: Supplementary file 1 [file ijms-24-08607-s001.zip › Supplementary Table S4.pdf]

Supplementary Table S4. Pathways associated with significantly altered proteins in bone marrow cells irradiated with 0.1Gy BM (A) and 3Gy (B) and common pathways associated with both doses. Pathway analysis was performed with Pathdip and Benjamini–Hochberg procedure (BH-method) was used to control FDR. FDR: false discovery rate

Table 4A<br>Pathways associated with deregulated proteins in the bone marrow cells of mice irradiated with 0.1Gy				
**Pathway Name**	**KEGG pathway class**	**KEGG pathway subclass**	**p-value**	**q-value (FDR: BH-method)**
Hepatitis C	6.Diseases-Infectious	6.3 Infectious disease: viral	2.36E-08	6.93E-06
Pathogenic Escherichia coli infection	6.Diseases-Infectious	6.4 Infectious disease: bacterial	1.26E-07	1.86E-05
Neurotrophin signaling	5. Organismal Systems	5.6 Nervous system	2.13E-07	2.09E-05
Apoptosis	4. Cellular Processes- Cell growth and death	4.2 Cell growth and death	1.13E-06	8.34E-05
Human immunodeficiency virus 1 infection	6.Diseases-Infectious	6.3 Infectious disease: viral	2.62E-06	1.54E-04
Proteoglycans in cancer	6.Diseases-cancer	6.1 Cancer: overview	5.02E-06	2.46E-04
Cholesterol metabolism	5. Organismal Systems	5.4 Digestive system	1.02E-05	4.28E-04
PI3K-Akt signaling	3. Environmental Information Processing- Signal transduction	3.2 Signal transduction	1.05E-05	3.86E-04
Influenza A	6.Diseases-Infectious	6.3 Infectious disease: viral	2.52E-05	8.24E-04
Legionellosis	6.Diseases-Infectious	6.4 Infectious disease: bacterial	2.66E-05	7.83E-04
Measles	6.Diseases-Infectious	6.3 Infectious disease: viral	3.48E-05	9.31E-04
Oxidative phosphorylation	1. Metabolism	1.2 Energy metabolism	3.74E-05	9.16E-04
Viral carcinogenesis	6.Diseases-cancer	6.1 Cancer: overview	4.12E-05	9.33E-04
Osteoclast differentiation	5. Organismal Systems	5.8 Development and regeneration	4.29E-05	9.00E-04
HIF-1 signaling	3. Environmental Information Processing- Signal transduction	3.2 Signal transduction	5.15E-05	1.01E-03
NF-kappa B signaling	3. Environmental Information Processing- Signal transduction	3.2 Signal transduction	5.58E-05	1.03E-03
Pathways in cancer	6.Diseases-cancer	6.1 Cancer: overview	6.21E-05	1.07E-03
T cell receptor signaling	5. Organismal Systems- Immune system	5.1 Immune system	6.49E-05	1.06E-03
Protein processing in endoplasmic reticulum	2. Genetic information processing	2.3 Folding, sorting and degradation	7.04E-05	1.09E-03
TNF signaling	3. Environmental Information Processing- Signal transduction	3.2 Signal transduction	1.16E-04	1.71E-03
Phagosome	4. Cellular Processes	4.1 Transport and catabolism	1.21E-04	1.69E-03
Human cytomegalovirus infection	6.Diseases-Infectious	6.3 Infectious disease: viral	1.23E-04	1.64E-03
Hepatitis B	6.Diseases-Infectious	6.3 Infectious disease: viral	1.67E-04	2.13E-03
Herpes simplex infection	6.Diseases-Infectious	6.3 Infectious disease: viral	1.68E-04	2.06E-03

ErbB signaling	3. Environmental Information Processing- Signal transduction	3.2 Signal transduction	1.83E-04	2.16E-03
Fluid shear stress and atherosclerosis	6.Diseases	6.9 Cardiovascular disease	2.09E-04	2.37E-03
Complement and coagulation cascades	5. Organismal Systems- Immune system	5.1 Immune system	2.57E-04	2.79E-03
Parkinson disease	6.Diseases	6.7 Neurodegenerative disease	2.57E-04	2.79E-03
IL-17 signaling	5. Organismal Systems- Immune system	5.1 Immune system	3.36E-04	3.41E-03
Prion diseases	6.Diseases	6.7 Neurodegenerative disease	3.42E-04	3.35E-03
Lysosome	4. Cellular Processes	4.1 Transport and catabolism	3.50E-04	3.32E-03
NOD-like receptor signaling	5. Organismal Systems- Immune system	5.1 Immune system	3.84E-04	3.53E-03
Citrate cycle (TCA cycle)	1. Metabolism	1.1 Carbohydrate metabolism	3.89E-04	3.46E-03
Central carbon metabolism in cancer	6.Diseases-cancer	6.1 Cancer: overview	4.08E-04	3.53E-03
Pertussis	6.Diseases-Infectious	6.4 Infectious disease: bacterial	4.15E-04	3.48E-03
Toxoplasmosis	6.Diseases-Infectious	6.5 Infectious disease: parasitic	5.32E-04	4.34E-03
Fc gamma R-mediated phagocytosis	5. Organismal Systems- Immune system	5.1 Immune system	5.50E-04	4.37E-03
Focal adhesion	4. Cellular Processes	4.3 Cellular community - eukaryotes	5.79E-04	4.48E-03
Bacterial invasion of epithelial cells	6.Diseases-Infectious	6.4 Infectious disease: bacterial	7.19E-04	5.42E-03
Human papillomavirus infection	6.Diseases-Infectious	6.3 Infectious disease: viral	7.92E-04	5.82E-03
Cell cycle	4. Cellular Processes- Cell growth and death	4.2 Cell growth and death	8.01E-04	5.74E-03
Adherens junction	4. Cellular Processes	4.3 Cellular community - eukaryotes	8.18E-04	5.73E-03
Non-alcoholic fatty liver disease (NAFLD)	6.Diseases	6.10 Endocrine and metabolic disease	9.07E-04	6.20E-03
Epithelial cell signaling in Helicobacter pylori infection	6.Diseases-Infectious	6.4 Infectious disease: bacterial	9.95E-04	6.65E-03
Tuberculosis	6.Diseases-Infectious	6.4 Infectious disease: bacterial	1.10E-03	7.22E-03
Amoebiasis	6.Diseases-Infectious	6.5 Infectious disease: parasitic	1.14E-03	7.31E-03
MicroRNAs in cancer	6.Diseases-cancer	6.1 Cancer: overview	1.15E-03	7.17E-03
Regulation of actin cytoskeleton	4. Cellular Processes	4.5 Cell motility	1.15E-03	7.07E-03
Hippo signaling	3. Environmental Information Processing- Signal transduction	3.2 Signal transduction	1.26E-03	7.55E-03
Huntington disease	6.Diseases	6.7 Neurodegenerative disease	1.29E-03	7.59E-03
mRNA surveillance	2. Genetic information processing	2.2 Translation	1.29E-03	7.46E-03
Tight junction	4. Cellular Processes	4.3 Cellular community - eukaryotes	1.30E-03	7.33E-03
Porphyrin and chlorophyll metabolism	1. Metabolism	1.8 Metabolism of cofactors and vitamins	1.31E-03	7.28E-03

Kaposi sarcoma-associated herpesvirus infection	6.Diseases-Infectious	6.3 Infectious disease: viral	1.58E-03	8.59E-03
MAPK signaling	3. Environmental Information Processing- Signal transduction	3.2 Signal transduction	1.75E-03	9.38E-03
Leishmaniasis	6.Diseases-Infectious	6.5 Infectious disease: parasitic	1.95E-03	1.03E-02
Spliceosome	2. Genetic information processing	2.1 Transcription	2.08E-03	1.07E-02
Ribosome	2. Genetic information processing	2.2 Translation	2.10E-03	1.07E-02
RNA degradation	2. Genetic information processing	2.3 Folding, sorting and degradation	2.25E-03	1.12E-02
Chagas disease (American trypanosomiasis)	6.Diseases-Infectious	6.5 Infectious disease: parasitic	2.30E-03	1.13E-02
Chemokine signaling	5. Organismal Systems-Immune system	5.1 Immune system	2.40E-03	1.16E-02
Salmonella infection	6.Diseases-Infectious	6.4 Infectious disease: bacterial	2.43E-03	1.15E-02
AGE-RAGE signaling pathway in diabetic complications	6.Diseases	6.10 Endocrine and metabolic disease	2.45E-03	1.14E-02
Endometrial cancer	6.Diseases-cancer	6.2 Cancer: specific types	2.57E-03	1.18E-02
Autophagy - animal	4. Cellular Processes	4.1 Transport and catabolism	2.64E-03	1.19E-02
Ras signaling	3. Environmental Information Processing- Signal transduction	3.2 Signal transduction	2.86E-03	1.27E-02
Fc epsilon RI signaling	5. Organismal Systems-Immune system	5.1 Immune system	2.96E-03	1.30E-02
Thyroid cancer	6.Diseases-cancer	6.2 Cancer: specific types	2.97E-03	1.29E-02
Staphylococcus aureus infection	6.Diseases-Infectious	6.4 Infectious disease: bacterial	2.97E-03	1.27E-02
Renal cell carcinoma	6.Diseases-cancer	6.2 Cancer: specific types	3.00E-03	1.26E-02
Primary immunodeficiency	6.Diseases- immune	6.6 Immune disease	3.62E-03	1.50E-02
Pancreatic cancer	6.Diseases-cancer	6.2 Cancer: specific types	3.78E-03	1.54E-02
Toll-like receptor signaling	5. Organismal Systems-Immune system	5.1 Immune system	3.84E-03	1.55E-02
Ubiquitin mediated proteolysis	2. Genetic information processing	2.3 Folding, sorting and degradation	3.89E-03	1.55E-02
B cell receptor signaling	5. Organismal Systems-Immune system	5.1 Immune system	3.99E-03	1.56E-02
Asthma	6.Diseases- immune	6.6 Immune disease	4.00E-03	1.55E-02
VEGF signaling	3. Environmental Information Processing- Signal transduction	3.2 Signal transduction	4.09E-03	1.56E-02
Propanoate metabolism	1. Metabolism	1.1 Carbohydrate metabolism	4.62E-03	1.74E-02
RNA polymerase	2. Genetic information processing	2.1 Transcription	4.78E-03	1.78E-02
Glutathione metabolism	1. Metabolism	1.6 Metabolism of other amino acids	5.18E-03	1.90E-02
Leukocyte transendothelial migration	5. Organismal Systems-Immune system	5.1 Immune system	6.29E-03	2.28E-02
Hematopoietic cell lineage	5. Organismal Systems-Immune system	5.1 Immune system	8.02E-03	2.88E-02

Platelet activation	5. Organismal Systems- Immune system	5.1 Immune system	8.47E-03	3.00E-02
Rap1 signaling	3. Environmental Information Processing- Signal transduction	3.2 Signal transduction	9.00E-03	3.15E-02
Th1 and Th2 cell differentiation	5. Organismal Systems- Immune system	5.1 Immune system	9.06E-03	3.13E-02
Epstein-Barr virus infection	6.Diseases-Infectious	6.3 Infectious disease: viral	9.15E-03	3.13E-02
PPAR signaling	5. Organismal Systems	5.2 Endocrine system	9.25E-03	3.12E-02
p53 signaling	4. Cellular Processes- Cell growth and death	4.2 Cell growth and death	9.87E-03	3.30E-02
RNA transport	2. Genetic information processing	2.2 Translation	1.02E-02	3.36E-02
Rheumatoid arthritis	6.Diseases- immune	6.6 Immune disease	1.06E-02	3.46E-02
Oocyte meiosis	4. Cellular Processes- Cell growth and death	4.2 Cell growth and death	1.08E-02	3.49E-02
Natural killer cell mediated cytotoxicity	5. Organismal Systems- Immune system	5.1 Immune system	1.10E-02	3.51E-02
Amyotrophic lateral sclerosis (ALS)	6.Diseases	6.7 Neurodegenerative disease	1.18E-02	3.74E-02
Human T-cell leukemia virus 1 infection	6.Diseases-Infectious	6.3 Infectious disease: viral	1.20E-02	3.74E-02
Pyruvate metabolism	1. Metabolism	1.1 Carbohydrate metabolism	1.23E-02	3.81E-02
Mitophagy - animal	4. Cellular Processes	4.1 Transport and catabolism	1.24E-02	3.78E-02
FoxO signaling	3. Environmental Information Processing- Signal transduction	3.2 Signal transduction	1.29E-02	3.90E-02
Vitamin digestion and absorption	5. Organismal Systems	5.4 Digestive system	1.39E-02	4.16E-02
RIG-I-like receptor signaling	5. Organismal Systems- Immune system	5.1 Immune system	1.48E-02	4.41E-02
Glycine, serine and threonine metabolism	1. Metabolism	1.5 Amino acid metabolism	1.53E-02	4.49E-02
Prostate cancer	6.Diseases-cancer	6.2 Cancer: specific types	1.55E-02	4.52E-02
Glyoxylate and dicarboxylate metabolism	1. Metabolism	1.1 Carbohydrate metabolism	1.67E-02	4.81E-02
Type I diabetes mellitus	6.Diseases	6.10 Endocrine and metabolic disease	1.72E-02	4.90E-02
Glycolysis / Gluconeogenesis	1. Metabolism	1.1 Carbohydrate metabolism	1.72E-02	4.86E-02
JAK-STAT signaling	3. Environmental Information Processing- Signal transduction	3.2 Signal transduction	1.72E-02	4.82E-02
Th17 cell differentiation	5. Organismal Systems- Immune system	5.1 Immune system	1.76E-02	4.87E-02
Linoleic acid metabolism	1. Metabolism	1.3 Lipid metabolism	1.85E-02	4.99E-02

Table 4B

Pathways associated with deregulated proteins in the bone marrow cells of mice irradiated with 3Gy

Pathway Name	KEGG pathway class	KEGG pathway subclass	p-value	q-value (FDR: BH-method)
RNA transport	2. Genetic information processing	2.2 Translation	1.6E-12	5.1E-10

Homologous recombination	2. Genetic information processing- Replication and repair	2.4 Replication and repair	4.2E-11	6.5E-09
Cell cycle	4. Cellular Processes- Cell growth and death	4.2 Cell growth and death	4.6E-11	4.8E-09
Spliceosome	2. Genetic information processing	2.1 Transcription	7.4E-11	5.7E-09
mRNA surveillance	2. Genetic information processing	2.2 Translation	1.3E-10	7.8E-09
DNA replication	2. Genetic information processing- Replication and repair	2.4 Replication and repair	2.0E-09	1.0E-07
Mismatch repair	2. Genetic information processing- Replication and repair	2.4 Replication and repair	4.2E-09	1.8E-07
RNA degradation	2. Genetic information processing	2.3 Folding, sorting and degradation	8.9E-09	3.4E-07
Viral carcinogenesis	6.Diseases-cancer	6.1 Cancer: overview	2.0E-08	6.7E-07
Ribosome biogenesis in eukaryotes	2. Genetic information processing	2.2 Translation	2.8E-08	8.7E-07
Huntington disease	6.Diseases	6.7 Neurodegenerative disease	4.3E-08	1.2E-06
Hepatitis C	6.Diseases-Infectious	6.3 Infectious disease: viral	5.3E-08	1.3E-06
Oocyte meiosis	4. Cellular Processes- Cell growth and death	4.2 Cell growth and death	6.1E-08	1.4E-06
Fanconi anemia	2. Genetic information processing- Replication and repair	2.4 Replication and repair	1.9E-07	4.3E-06
Nucleotide excision repair	2. Genetic information processing- Replication and repair	2.4 Replication and repair	1.2E-06	2.6E-05
Neurotrophin signaling	5. Organismal Systems	5.6 Nervous system	1.8E-06	3.5E-05
Apoptosis	4. Cellular Processes- Cell growth and death	4.2 Cell growth and death	2.5E-06	4.6E-05
Pathogenic Escherichia coli infection	6.Diseases-Infectious	6.4 Infectious disease: bacterial	5.6E-06	9.5E-05
Pyrimidine metabolism	1. Metabolism	1.4 Nucleotide metabolism	9.3E-06	1.5E-04
PI3K-Akt signaling	3. Environmental Information Processing- Signal transduction	3.2 Signal transduction	1.3E-05	2.0E-04
RNA polymerase	2. Genetic information processing	2.1 Transcription	1.6E-05	2.4E-04
Herpes simplex infection	6.Diseases-Infectious	6.3 Infectious disease: viral	1.9E-05	2.6E-04
Glyoxylate and dicarboxylate metabolism	1. Metabolism	1.1 Carbohydrate metabolism	2.0E-05	2.6E-04
Purine metabolism	1. Metabolism	1.4 Nucleotide metabolism	2.5E-05	3.3E-04
Biosynthesis of unsaturated fatty acids	1. Metabolism	1.3 Lipid metabolism	2.9E-05	3.6E-04
Hepatitis B	6.Diseases-Infectious	6.3 Infectious disease: viral	5.4E-05	6.4E-04
Thermogenesis	5. Organismal Systems	5.10 Environmental adaptation	7.8E-05	8.8E-04
Autophagy - other	4. Cellular Processes	4.1 Transport and catabolism	8.6E-05	9.4E-04
Thyroid cancer	6.Diseases-cancer	6.2 Cancer: specific types	2.0E-04	2.1E-03

MicroRNAs in cancer	6.Diseases-cancer	6.1 Cancer: overview	3.2E-04	3.2E-03
Tight junction	4. Cellular Processes	4.3 Cellular community - eukaryotes	3.3E-04	3.2E-03
Ubiquitin mediated proteolysis	2. Genetic information processing	2.3 Folding, sorting and degradation	3.5E-04	3.4E-03
Basal transcription factors	2. Genetic information processing	2.1 Transcription	3.5E-04	3.3E-03
Pentose phosphate	1. Metabolism	1.1 Carbohydrate metabolism	3.7E-04	3.4E-03
Renal cell carcinoma	6.Diseases-cancer	6.2 Cancer: specific types	4.3E-04	3.7E-03
Hippo signaling	3. Environmental Information Processing- Signal transduction	3.2 Signal transduction	5.2E-04	4.4E-03
Oxidative phosphorylation	1. Metabolism	1.2 Energy metabolism	6.9E-04	5.7E-03
Pertussis	6.Diseases-Infectious	6.4 Infectious disease: bacterial	7.8E-04	6.3E-03
ErbB signaling	3. Environmental Information Processing- Signal transduction	3.2 Signal transduction	9.2E-04	7.3E-03
NF-kappa B signaling	3. Environmental Information Processing- Signal transduction	3.2 Signal transduction	9.7E-04	7.5E-03
Central carbon metabolism in cancer	6.Diseases-cancer	6.1 Cancer: overview	1.0E-03	7.5E-03
Pyruvate metabolism	1. Metabolism	1.1 Carbohydrate metabolism	1.0E-03	7.4E-03
p53 signaling	4. Cellular Processes- Cell growth and death	4.2 Cell growth and death	1.1E-03	8.1E-03
Human immunodeficiency virus 1 infection	6.Diseases-Infectious	6.3 Infectious disease: viral	1.2E-03	8.2E-03
Fc gamma R-mediated phagocytosis	5. Organismal Systems- Immune system	5.1 Immune system	1.2E-03	8.1E-03
Ribosome	2. Genetic information processing	2.2 Translation	1.5E-03	9.8E-03
Citrate cycle (TCA cycle)	1. Metabolism	1.1 Carbohydrate metabolism	1.6E-03	1.0E-02
Human T-cell leukemia virus 1 infection	6.Diseases-Infectious	6.3 Infectious disease: viral	1.7E-03	1.1E-02
Bacterial invasion of epithelial cells	6.Diseases-Infectious	6.4 Infectious disease: bacterial	1.8E-03	1.1E-02
Salmonella infection	6.Diseases-Infectious	6.4 Infectious disease: bacterial	1.9E-03	1.2E-02
Proteoglycans in cancer	6.Diseases-cancer	6.1 Cancer: overview	2.0E-03	1.2E-02
Legionellosis	6.Diseases-Infectious	6.4 Infectious disease: bacterial	2.2E-03	1.3E-02
Influenza A	6.Diseases-Infectious	6.3 Infectious disease: viral	2.2E-03	1.3E-02
Colorectal cancer	6.Diseases-cancer	6.2 Cancer: specific types	2.5E-03	1.4E-02
NOD-like receptor signaling	5. Organismal Systems- Immune system	5.1 Immune system	2.5E-03	1.4E-02
Mitophagy - animal	4. Cellular Processes	4.1 Transport and catabolism	2.5E-03	1.4E-02
Non-alcoholic fatty liver disease (NAFLD)	6.Diseases	6.10 Endocrine and metabolic disease	2.5E-03	1.4E-02
Peroxisome	4. Cellular Processes	4.1 Transport and catabolism	2.7E-03	1.4E-02

HIF-1 signaling	3. Environmental Information Processing- Signal transduction	3.2 Signal transduction	2.8E-03	1.5E-02
Cholesterol metabolism	5. Organismal Systems	5.4 Digestive system	3.1E-03	1.6E-02
Cellular senescence	4. Cellular Processes- Cell growth and death	4.2 Cell growth and death	3.2E-03	1.6E-02
Human cytomegalovirus infection	6.Diseases-Infectious	6.3 Infectious disease: viral	3.4E-03	1.7E-02
B cell receptor signaling	5. Organismal Systems- Immune system	5.1 Immune system	3.5E-03	1.7E-02
Aminoacyl-tRNA biosynthesis	2. Genetic information processing	2.2 Translation	3.5E-03	1.7E-02
IL-17 signaling	5. Organismal Systems- Immune system	5.1 Immune system	4.2E-03	2.0E-02
Propanoate metabolism	1. Metabolism	1.1 Carbohydrate metabolism	4.4E-03	2.0E-02
Thyroid hormone signaling	5. Organismal Systems	5.2 Endocrine system	4.4E-03	2.0E-02
TNF signaling	3. Environmental Information Processing- Signal transduction	3.2 Signal transduction	5.0E-03	2.3E-02
AGE-RAGE signaling pathway in diabetic complications	6.Diseases	6.10 Endocrine and metabolic disease	5.3E-03	2.4E-02
Glycine, serine and threonine metabolism	1. Metabolism	1.5 Amino acid metabolism	5.4E-03	2.3E-02
Glycolysis / Gluconeogenesis	1. Metabolism	1.1 Carbohydrate metabolism	5.4E-03	2.3E-02
Fluid shear stress and atherosclerosis	6.Diseases	6.9 Cardiovascular disease	6.3E-03	2.7E-02
Fructose and mannose metabolism	1. Metabolism	1.1 Carbohydrate metabolism	6.5E-03	2.8E-02
T cell receptor signaling	5. Organismal Systems- Immune system	5.1 Immune system	6.6E-03	2.7E-02
Glycosaminoglycan degradation	1. Metabolism	1.7 Glycan biosynthesis and metabolism	6.6E-03	2.7E-02
Other glycan degradation	1. Metabolism	1.7 Glycan biosynthesis and metabolism	6.6E-03	2.7E-02
Osteoclast differentiation	5. Organismal Systems	5.8 Development and regeneration	7.8E-03	3.1E-02
Amyotrophic lateral sclerosis (ALS)	6.Diseases	6.7 Neurodegenerative disease	8.0E-03	3.1E-02
Parkinson disease	6.Diseases	6.7 Neurodegenerative disease	8.0E-03	3.1E-02
Shigellosis	6.Diseases-Infectious	6.4 Infectious disease: bacterial	8.4E-03	3.2E-02
Lysosome	4. Cellular Processes	4.1 Transport and catabolism	9.0E-03	3.4E-02
Kaposi sarcoma-associated herpesvirus infection	6.Diseases-Infectious	6.3 Infectious disease: viral	9.7E-03	3.6E-02
Sphingolipid signaling	3. Environmental Information Processing- Signal transduction	3.2 Signal transduction	1.0E-02	3.7E-02
Pathways in cancer	6.Diseases-cancer	6.1 Cancer: overview	1.0E-02	3.8E-02
Porphyrin and chlorophyll metabolism	1. Metabolism	1.8 Metabolism of cofactors and vitamins	1.1E-02	3.9E-02
Acute myeloid leukemia	6.Diseases-cancer	6.2 Cancer: specific types	1.2E-02	4.3E-02

Glutathione metabolism	1. Metabolism	1.6 Metabolism of other amino acids	1.3E-02	4.7E-02

Table 4C
Common pathways associated with deregulated proteins in the bone marrow cells of mice irradiated with 0.1Gy and 3Gy

Pathway Name	KEGG pathway class	KEGG pathway subclass	Group			
			BM 0.1Gy		BM 3Gy	
			p-value	q-value (FDR: BH-method)	p-value	q-value (FDR: BH-method)
Citrate cycle (TCA cycle)	1. Metabolism	1.1 Carbohydrate metabolism	3.89E-04	3.46E-03	1.56E-03	1.02E-02
Glycolysis / Gluconeogenesis	1. Metabolism	1.1 Carbohydrate metabolism	1.72E-02	4.86E-02	5.41E-03	2.34E-02
Glyoxylate and dicarboxylate metabolism	1. Metabolism	1.1 Carbohydrate metabolism	1.67E-02	4.81E-02	1.96E-05	2.61E-04
Propanoate metabolism	1. Metabolism	1.1 Carbohydrate metabolism	4.62E-03	1.74E-02	4.36E-03	2.03E-02
Pyruvate metabolism	1. Metabolism	1.1 Carbohydrate metabolism	1.23E-02	3.81E-02	1.01E-03	7.37E-03
Oxidative phosphorylation	1. Metabolism	1.2 Energy metabolism	3.74E-05	9.16E-04	6.90E-04	5.73E-03
Glycine, serine and threonine metabolism	1. Metabolism	1.5 Amino acid metabolism	1.53E-02	4.49E-02	5.36E-03	2.35E-02
Glutathione metabolism	1. Metabolism	1.6 Metabolism of other amino acids	5.18E-03	1.90E-02	1.33E-02	4.71E-02
Porphyrin and chlorophyll metabolism	1. Metabolism	1.8 Metabolism of cofactors and vitamins	1.31E-03	7.28E-03	1.07E-02	3.85E-02
RNA polymerase	2. Genetic information processing	2.1 Transcription	4.78E-03	1.78E-02	1.64E-05	2.39E-04
Spliceosome	2. Genetic information processing	2.1 Transcription	2.08E-03	1.07E-02	7.40E-11	5.68E-09
mRNA surveillance	2. Genetic information processing	2.2 Translation	1.29E-03	7.46E-03	1.28E-10	7.84E-09
Ribosome	2. Genetic information processing	2.2 Translation	2.10E-03	1.07E-02	1.47E-03	9.79E-03
RNA transport	2. Genetic information processing	2.2 Translation	1.02E-02	3.36E-02	1.65E-12	5.06E-10
RNA degradation	2. Genetic information processing	2.3 Folding, sorting and degradation	2.25E-03	1.12E-02	8.90E-09	3.42E-07
Ubiquitin mediated proteolysis	2. Genetic information processing	2.3 Folding, sorting and degradation	3.89E-03	1.55E-02	3.52E-04	3.38E-03

ErbB signaling	3. Environmental Information Processing- Signal transduction	3.2 Signal transduction	1.83E-04	2.16E-03	9.23E-04	7.27E-03
HIF-1 signaling	3. Environmental Information Processing- Signal transduction	3.2 Signal transduction	5.15E-05	1.01E-03	2.83E-03	1.47E-02
Hippo signaling	3. Environmental Information Processing- Signal transduction	3.2 Signal transduction	1.26E-03	7.55E-03	5.18E-04	4.42E-03
NF-kappa B signaling	3. Environmental Information Processing- Signal transduction	3.2 Signal transduction	5.58E-05	1.03E-03	9.71E-04	7.45E-03
PI3K-Akt signaling	3. Environmental Information Processing- Signal transduction	3.2 Signal transduction	1.05E-05	3.86E-04	1.33E-05	2.04E-04
TNF signaling	3. Environmental Information Processing- Signal transduction	3.2 Signal transduction	1.16E-04	1.71E-03	5.05E-03	2.28E-02
Lysosome	4. Cellular Processes	4.1 Transport and catabolism	3.50E-04	3.32E-03	9.01E-03	3.41E-02
Mitophagy - animal	4. Cellular Processes	4.1 Transport and catabolism	1.24E-02	3.78E-02	2.52E-03	1.38E-02
Apoptosis	4. Cellular Processes- Cell growth and death	4.2 Cell growth and death	1.13E-06	8.34E-05	2.54E-06	4.59E-05
Cell cycle	4. Cellular Processes- Cell growth and death	4.2 Cell growth and death	8.01E-04	5.74E-03	4.65E-11	4.75E-09
Oocyte meiosis	4. Cellular Processes- Cell growth and death	4.2 Cell growth and death	1.08E-02	3.49E-02	6.08E-08	1.44E-06
p53 signaling	4. Cellular Processes- Cell growth and death	4.2 Cell growth and death	9.87E-03	3.30E-02	1.14E-03	8.14E-03
Tight junction	4. Cellular Processes	4.3 Cellular community - eukaryotes	1.30E-03	7.33E-03	3.25E-04	3.22E-03
B cell receptor signaling	5. Organismal Systems-Immune system	5.1 Immune system	3.99E-03	1.56E-02	3.50E-03	1.71E-02
Fc gamma R-mediated phagocytosis	5. Organismal Systems-Immune system	5.1 Immune system	5.50E-04	4.37E-03	1.19E-03	8.12E-03
IL-17 signaling	5. Organismal Systems-Immune system	5.1 Immune system	3.36E-04	3.41E-03	4.20E-03	1.98E-02
NOD-like receptor signaling	5. Organismal Systems-Immune system	5.1 Immune system	3.84E-04	3.53E-03	2.50E-03	1.40E-02
T cell receptor signaling	5. Organismal Systems-Immune system	5.1 Immune system	6.49E-05	1.06E-03	6.59E-03	2.73E-02
Cholesterol metabolism	5. Organismal Systems	5.4 Digestive system	1.02E-05	4.28E-04	3.08E-03	1.58E-02

Neurotrophin signaling	5. Organismal Systems	5.6 Nervous system	2.13E-07	2.09E-05	1.82E-06	3.48E-05
Osteoclast differentiation	5. Organismal Systems	5.8 Development and regeneration	4.29E-05	9.00E-04	7.84E-03	3.13E-02
Central carbon metabolism in cancer	6.Diseases-cancer	6.1 Cancer: overview	4.08E-04	3.53E-03	1.01E-03	7.53E-03
MicroRNAs in cancer	6.Diseases-cancer	6.1 Cancer: overview	1.15E-03	7.17E-03	3.17E-04	3.24E-03
Pathways in cancer	6.Diseases-cancer	6.1 Cancer: overview	6.21E-05	1.07E-03	1.05E-02	3.83E-02
Proteoglycans in cancer	6.Diseases-cancer	6.1 Cancer: overview	5.02E-06	2.46E-04	1.97E-03	1.18E-02
Viral carcinogenesis	6.Diseases-cancer	6.1 Cancer: overview	4.12E-05	9.33E-04	1.97E-08	6.72E-07
AGE-RAGE signaling pathway in diabetic complications	6.Diseases	6.10 Endocrine and metabolic disease	2.45E-03	1.14E-02	5.30E-03	2.36E-02
Non-alcoholic fatty liver disease (NAFLD)	6.Diseases	6.10 Endocrine and metabolic disease	9.07E-04	6.20E-03	2.53E-03	1.36E-02
Renal cell carcinoma	6.Diseases-cancer	6.2 Cancer: specific types	3.00E-03	1.26E-02	4.27E-04	3.75E-03
Thyroid cancer	6.Diseases-cancer	6.2 Cancer: specific types	2.97E-03	1.29E-02	2.03E-04	2.15E-03
Hepatitis B	6.Diseases-Infectious	6.3 Infectious disease: viral	1.67E-04	2.13E-03	5.38E-05	6.36E-04
Hepatitis C	6.Diseases-Infectious	6.3 Infectious disease: viral	2.36E-08	6.93E-06	5.25E-08	1.34E-06
Herpes simplex infection	6.Diseases-Infectious	6.3 Infectious disease: viral	1.68E-04	2.06E-03	1.88E-05	2.62E-04
Human cytomegalovirus infection	6.Diseases-Infectious	6.3 Infectious disease: viral	1.23E-04	1.64E-03	3.43E-03	1.70E-02
Human immunodeficiency virus 1 infection	6.Diseases-Infectious	6.3 Infectious disease: viral	2.62E-06	1.54E-04	1.18E-03	8.21E-03
Human T-cell leukemia virus 1 infection	6.Diseases-Infectious	6.3 Infectious disease: viral	1.20E-02	3.74E-02	1.73E-03	1.11E-02
Influenza A	6.Diseases-Infectious	6.3 Infectious disease: viral	2.52E-05	8.24E-04	2.24E-03	1.30E-02
Kaposi sarcoma-associated herpesvirus infection	6.Diseases-Infectious	6.3 Infectious disease: viral	1.58E-03	8.59E-03	9.71E-03	3.63E-02
Bacterial invasion of epithelial cells	6.Diseases-Infectious	6.4 Infectious disease: bacterial	7.19E-04	5.42E-03	1.80E-03	1.12E-02
Legionellosis	6.Diseases-Infectious	6.4 Infectious disease: bacterial	2.66E-05	7.83E-04	2.22E-03	1.31E-02
Pathogenic Escherichia coli infection	6.Diseases-Infectious	6.4 Infectious disease: bacterial	1.26E-07	1.86E-05	5.57E-06	9.50E-05
Pertussis	6.Diseases-Infectious	6.4 Infectious disease: bacterial	4.15E-04	3.48E-03	7.81E-04	6.31E-03
Salmonella infection	6.Diseases-Infectious	6.4 Infectious disease: bacterial	2.43E-03	1.15E-02	1.93E-03	1.18E-02
Amyotrophic lateral sclerosis (ALS)	6.Diseases-Infectious	6.7 Neurodegenerative disease	1.18E-02	3.74E-02	7.98E-03	3.14E-02
Huntington disease	6.Diseases-Infectious	6.7 Neurodegenerative disease	1.29E-03	7.59E-03	4.26E-08	1.19E-06
Parkinson disease	6.Diseases-Infectious	6.7 Neurodegenerative disease	2.57E-04	2.79E-03	7.98E-03	3.10E-02
Fluid shear stress and atherosclerosis	6.Diseases	6.9 Cardiovascular disease	2.09E-04	2.37E-03	6.33E-03	2.70E-02
