# Supplementary material for: The miRNA Content of Bone Marrow-Derived Extracellular Vesicles Contributes to Protein Pathway Alterations Involved in Ionising Radiation-Induced Bystander Responses
Source: Int J Mol Sci. 2023 May 11;24(10):8607. doi: 10.3390/ijms24108607 (PMC10218377; doi:10.3390/ijms24108607)
Supplement: Supplementary file 1 [file ijms-24-08607-s001.zip › Supplementary Table S5.pdf]

Supplementary Table S5. Significantly altered proteins in the bone marrow cells of mice treated with bone marrow-derived extracellular vesicles from mice irradiated with 0.1Gy (A) and 3Gy (B) compared to treatment with extracellular vesicles from control mice and common proteins between the two treatment groups (C). The number of PSM's is the total number of identified peptide spectra matched for the protein (PSM- peptide spectrum matches).

Table 5A<br>Deregulated proteins in the bone marrow cells of mice treated with extracellular vesicles from mice irradiated with 0.1Gy						
**BM+0.1Gy EV downregulated proteins**						
**Protein name**	**Gene Symbol**	**UniProt ID**	**PSMs**	**Unique Peptides**	**Abundance Ratio 0.1Gy vs 0Gy**	**Abundance Ratio Adj. P-Value: 0.1Gy vs 0Gy**
FAS-associated factor 2	Faf2	Q3TDN2	5	3	0.01	5.57E-16
Histone acetyltransferase p300	Ep300	B2RWS6	2	2	0.01	5.57E-16
TIP41-like protein	Tiprl	Q8BH58	3	2	0.01	5.57E-16
Sorting nexin-29	Snx29	Q9D3S3	2	2	0.182	5.57E-16
Protein phosphatase 1 regulatory subunit 14B	Ppp1r14b	Q62084	19	2	0.328	1.23E-04
p21-activated protein kinase-interacting protein 1	Pak1ip1	Q9DCE5	7	2	0.382	1.36E-08
Beta-galactosidase	Glb1	P23780	4	2	0.481	3.20E-06
Thioredoxin. mitochondrial	Txn2	P97493	6	2	0.518	2.91E-03
Renin receptor	Atp6ap2	Q9CYN9	5	2	0.532	7.96E-03
Huntingtin-interacting protein K	2310003F16Rik; Hypk	Q9CR41	4	2	0.534	1.19E-02
Exosome complex exonuclease RRP44	Dis3	Q9CSH3	3	2	0.55	5.38E-05
Prostaglandin reductase-3	Zadh2	Q8BGC4	4	2	0.619	3.26E-03
Carbonyl reductase [NADPH] 1	Cbr1	P48758	17	5	0.631	1.39E-03
Integrin alpha-1	Itga1	Q3V3R4	7	4	0.642	3.82E-02
RNA-binding protein FUS	Fus	P56959	43	4	0.644	2.05E-03
Low molecular weight phosphotyrosine protein phosphatase	Acp1; LOC631286; LOC102642088	Q9D358	57	4	0.649	2.12E-04
Cathepsin Z	Ctsz	Q9WUU7	82	4	0.654	2.51E-06
Pro-cathepsin H	Ctsh	P49935	25	4	0.664	6.47E-03
RNA-binding protein 3	Rbm3	O89086	165	5	0.668	5.19E-05
Armadillo repeat-containing protein 6	Armc6	Q8BNU0	5	2	0.669	1.30E-02
Monocyte differentiation antigen CD14	Cd14	P10810	7	2	0.682	1.37E-02
Lysosomal protective protein	Ctsa	P16675	36	4	0.691	1.76E-02
TBC1 domain family member 15	Tbc1d15	Q9CXF4	36	6	0.692	1.57E-02

Glycogen phosphorylase. muscle form	Pygm	Q9WUB 3	533	4	0.697	4.40E-02
Chloride intracellular channel protein 4	Clic4	Q9QYB1	57	5	0.697	2.25E-02
U4/U6 small nuclear ribonucleoprotein Prp31	Prpf31	Q8CCF0	16	3	0.702	2.51E-02
Annexin A4	Anxa4	P97429	178	10	0.705	4.25E-03
40S ribosomal protein S11	Rps11	P62281	108	7	0.718	1.66E-03
Fatty acid-binding protein. adipocyte	Fabp4	P04117	38	5	0.721	4.64E-02
LIM and SH3 domain protein 1	Lasp1	Q61792	71	7	0.733	2.15E-02
Very-long-chain (3R)-3-hydroxyacyl-CoA dehydratase 2	Ptplb; Hacd2	Q9D3B1	35	3	0.744	3.96E-02
40S ribosomal protein S14	Rps14	P62264	153	5	0.752	1.14E-02
High mobility group protein HMG-I/HMG-Y	Hmga1; Hmga1-rs1; Hmga1b	P17095	130	2	0.77	1.66E-02

BM+0.1Gy EV upregulated proteins

Protein name	Gene Symbol	UniProt ID	PSMs	Unique Peptides	Abundance Ratio 0.1Gy vs 0Gy	Abundance Ratio Adj. P-Value: 0.1Gy vs 0Gy
Protein POF1B	Pof1b	Q8K4L4	4	2	15.454	5.57E-16
Trans-2-enoyl-CoA reductase, mitochondrial	Mecr	Q9DCS3	3	2	100	5.57E-16
Carbonyl reductase [NADPH] 2	Cbr2	P08074	2	2	100	5.57E-16
RUN and FYVE domain-containing protein 1	Rufy1	Q8BIJ7	5	2	100	5.57E-16
Sphingolipid delta(4)-desaturase DES1	Degs1	O09005	5	2	100	5.57E-16
Myosin regulatory light polypeptide 9	Myl9	Q9CQ19	197	2	100	5.57E-16
Cysteine protease ATG4B	Atg4b	Q8BGE6	6	2	100	5.57E-16
Vacuolar protein sorting-associated protein 18 homolog	Vps18	Q8R307	7	2	4.868	3.30E-08
Myosin-4	Myh4	Q5SX39	291	10	1.553	4.23E-07
Complement component 1 Q subcomponent-binding protein, mitochondrial	C1qbp	O35658	270	7	1.55	1.02E-06
Butyrophilin-like protein 10	Btnl10	Q9JK39	15	4	2.322	6.45E-06
Protein PRRC2C	Prrc2c	Q3TLH4	11	2	1.495	1.49E-05
Cytochrome c oxidase subunit 2	COX2	P00405	606	5	1.493	1.66E-05
H-2 class II histocompatibility antigen, A-K alpha chain	H2-Aa	P01910	100	6	1.624	4.92E-05
Keratin, type II cuticular Hb4	Krt84	Q99M73	391	2	1.852	5.77E-05
Cytochrome b5	Cyb5; Cyb5a	P56395	145	5	1.4	1.85E-04
Cytochrome b-c1 complex subunit 6, mitochondrial	Uqcrh	P99028	73	2	1.715	2.29E-04

Platelet factor 4	Pf4	Q9Z126	307	3	1.401	3.52E-04
Myosin light chain 1/3, skeletal muscle isoform	Myl1	P05977	47	5	1.721	5.59E-04
Ig alpha chain C region	Igh-VJ558	P01878	90	7	1.559	6.09E-04
Integrin alpha-IIb	Itga2b	Q9QUM 0	657	24	1.395	6.43E-04
N-acetylglucosamine-1-phosphodiester alpha-N-acetylglucosaminidase	Nagpa	Q8BJ48	3	2	1.972	6.56E-04
Cytochrome b-c1 complex subunit 7	Uqcrb	Q9D855	146	4	1.391	6.74E-04
ADP-ribosyl cyclase/cyclic ADP-ribose hydrolase 1	Cd38	P56528	59	4	1.666	8.33E-04
Histone H1.0	H1f0	P10922	184	4	1.351	9.27E-04
Peroxiredoxin-4	Prdx4	O08807	230	6	1.513	1.11E-03
Ferrochelatase, mitochondrial	Fech	P22315	303	15	1.374	1.32E-03
Nucleobindin-1	Nucb1	Q02819	93	10	1.436	1.34E-03
Cell cycle control protein 50A	Tmem30a	Q8VEK0	127	5	1.372	1.40E-03
MICOS complex subunit Mic27	Apool	Q78IK4	79	7	1.362	1.54E-03
Mitochondrial import inner membrane translocase subunit Tim8 A	Timm8a1	Q9WVA 2	9	2	1.625	2.34E-03
Propionyl-CoA carboxylase alpha chain, mitochondrial	Pcca	Q91ZA3	7	3	1.831	2.36E-03
Protein ERGIC-53	Lman1	Q9D0F3	196	9	1.357	2.39E-03
Mitochondrial 2-oxodicarboxylate carrier	Slc25a21	Q8BZ09	15	3	1.795	2.60E-03
NADH dehydrogenase [ubiquinone] flavoprotein 1, mitochondrial	Ndufv1	Q91YT0	174	12	1.333	2.74E-03
Keratin, type II cytoskeletal 71	Krt71	Q9R0H5	1161	2	1.96	3.18E-03
Ras-related protein Ral-A	Rala	P63321	90	2	1.902	3.23E-03
F-box only protein 50	Nccrp1	G3X9C2	6	3	2.307	3.40E-03
Anoctamin-6	Ano6	Q6P9J9	26	7	1.596	3.98E-03
Electron transfer flavoprotein-ubiquinone oxidoreductase, mitochondrial	Etfdh	Q921G7	184	16	1.446	4.25E-03
CDGSH iron-sulfur domain-containing protein 1	Cisd1	Q91WS0	42	3	1.567	5.49E-03
Histone H3-like centromeric protein A	Cenpa	O35216	11	2	1.575	6.12E-03
CD48 antigen	Cd48	P18181	84	5	1.485	6.73E-03
Histone H1.1	Hist1h1a	P43275	1596	4	1.321	7.36E-03
Calumenin	Calu	O35887	11	2	1.426	7.45E-03
Alpha-N-acetylneuraminide alpha-2,8-sialyltransferase	St8sia1	Q64687	4	2	1.311	8.04E-03
Integrin beta-3	Itgb3	O54890	538	21	1.315	8.78E-03

Prosaposin	Psap	Q61207	482	13	1.315	8.78E-03
Serotransferrin	Trf	Q921I1	1154	33	1.314	9.04E-03
Transmembrane emp24 domain-containing protein 10	Tmed10	Q9D1D4	282	6	1.306	9.68E-03
Histone H2B type 3-A	Hist3h2ba	Q9D2U9	8208	2	1.567	9.68E-03
H-2 class I histocompatibility antigen, K-K alpha chain	H2-K1	P04223	409	4	1.396	1.01E-02
Cytochrome b-c1 complex subunit 2, mitochondrial	Uqcrc2	Q9DB77	414	19	1.307	1.10E-02
Cytochrome c1, heme protein, mitochondrial	Cyc1	Q9D0M3	163	5	1.302	1.11E-02
Thioredoxin domain-containing protein 5	Txndc5	Q91W90	203	11	1.349	1.12E-02
GrpE protein homolog 1, mitochondrial	Grpel1	Q99LP6	105	7	1.401	1.14E-02
H-2 class II histocompatibility antigen, A-K beta chain	H2-Ab1	P06343	129	7	1.405	1.22E-02
Cold-inducible RNA-binding protein	Cirbp	P60824	3	2	2.016	1.62E-02
Membrane-associated progesterone receptor component 2	Pgrmc2	Q80UU9	150	5	1.325	2.25E-02
Transmembrane emp24 domain-containing protein 9	Tmed9	Q99KF1	125	4	1.322	2.60E-02
Cathepsin E	Ctse	P70269	156	3	1.329	2.79E-02
H-2 class II histocompatibility antigen, I-A beta chain	H2-Eb1	P18468	61	3	1.466	2.80E-02
ATP synthase subunit g, mitochondrial	Atp5l	Q9CPQ8	141	2	1.318	2.85E-02
Protein RER1	Rer1	Q9CQU3	39	4	1.452	3.18E-02
Enoyl-CoA delta isomerase 1, mitochondrial	Eci1	P42125	121	7	1.328	3.73E-02
Keratin, type I cuticular Ha1	Krt31	Q61765	521	4	1.514	3.98E-02
Platelet glycoprotein IX	Gp9	O88186	22	2	1.452	4.08E-02
Acylcarnitine hydrolase	Ces2c	Q91WG0	81	5	1.36	4.20E-02
Alpha-1,3-mannosyl-glycoprotein 2-beta-N-acetylglucosaminyltransferase	Mgat1	P27808	3	3	2.037	4.36E-02
Calcineurin B homologous protein 1	1500003O03Rik; Chp1	P61022	52	5	1.33	4.38E-02
C-type lectin domain family 1 member B	Clec1b	Q9JL99	25	2	1.447	4.40E-02
Protein PRRC1	Prrc1	Q3UPH1	33	5	1.525	4.67E-02
Equilibrative nucleoside transporter 1	Slc29a1	Q9JIM1	66	4	1.423	4.77E-02

Table 5B<br>Deregulated proteins in the bone marrow cells of mice treated with extracellular vesicles from mice irradiated with 3Gy						
**BM+3Gy EV downregulated proteins**						
**Protein name**	**Gene Symbol**	**UniProt ID**	**PSMs**	**Unique Peptides**	**Abundance Ratio 3Gy vs 0Gy**	**Abundance Ratio Adj. P-Value: 3Gy vs 0Gy**
TIP41-like protein	Tiprl	Q8BH58	3	2	0.01	4.51E-16
Nesprin-3	4831426I19Rik; Syne3	Q4FZC9	8	2	0.01	4.51E-16
RNA polymerase-associated protein RTF1 homolog	Rtf1	A2AQ19	2	2	0.01	4.51E-16
p21-activated protein kinase-interacting protein 1	Pak1ip1	Q9DCE5	7	2	0.01	4.51E-16
Translation initiation factor eIF-2B subunit alpha	Eif2b1	Q99LC8	3	2	0.01	4.51E-16
Galectin-related protein	Lgalsl	Q8VED9	3	2	0.224	2.18E-05
Ubiquitin-associated domain-containing protein 1	Ubac1	Q8VDI7	30	3	0.329	4.97E-03
N-acylneuraminate-9-phosphatase	Nanp	Q9CPT3	8	2	0.331	5.32E-03
Ubiquitin-conjugating enzyme E2 C	Ube2c	Q9D1C1	5	3	0.386	3.96E-04
Uroporphyrinogen-III synthase	Uros	P51163	56	7	0.391	1.77E-05
DnaJ homolog subfamily B member 6	Dnajb6	O54946	4	2	0.431	3.74E-02
Alpha-synuclein	Snca	O55042	92	4	0.435	1.99E-04
Alpha-hemoglobin-stabilizing protein	Ahsp	Q9CY02	318	6	0.464	6.87E-07
Tubulin--tyrosine ligase-like protein 12	Ttll12	Q3UDE2	96	12	0.479	1.08E-03
Cellular nucleic acid-binding protein	Cnbp	P53996	30	2	0.479	4.24E-02
Bisphosphoglycerate mutase	Bpgm	P15327	643	14	0.48	4.34E-07
Glycogen synthase kinase-3 alpha	Gsk3a	Q2NL51	25	2	0.482	4.60E-06
Chloride intracellular channel protein 4	Clic4	Q9QYB1	57	5	0.491	4.58E-03
High affinity immunoglobulin epsilon receptor subunit gamma	Fcer1g	P20491	20	2	0.498	3.00E-05
U4/U6 small nuclear ribonucleoprotein Prp31	Prpf31	Q8CCF0	16	3	0.516	1.17E-02
Carbonyl reductase [NADPH] 1	Cbr1	P48758	17	5	0.548	2.68E-02
Delta-aminolevulinic acid dehydratase	Alad	P10518	1021	12	0.572	1.46E-03
Hemoglobin subunit beta-1	Hbb-b1; Beta-s; Hbb-bs; Hbb-bt	P02088	47412	14	0.576	1.80E-03
Hemoglobin subunit beta-2	Hbb-b2	P02089	40357	11	0.594	4.19E-03
Carbonic anhydrase 1	Car1	P13634	2432	14	0.594	4.30E-03
Protein-glutamine gamma-glutamyltransferase 2	Tgm2	P21981	96	12	0.603	4.22E-03

Eosinophil cationic protein 1	Ear1; LOC101056653	P97426	849	5	0.644	3.18E-02
Carbonic anhydrase 2	Car2	P00920	4434	16	0.644	3.18E-02
Hemoglobin subunit alpha	Hba-a1; Hba-a2	P01942	49649	17	0.653	4.33E-02

BM+3Gy EV upregulated proteins

**Protein name**	**Gene Symbol**	**UniProt ID**	**PSMs**	**Unique Peptides**	**Abundance Ratio 3Gy vs 0Gy**	**Abundance Ratio Adj. P-Value: 3Gy vs 0Gy**
Carbonyl reductase [NADPH] 2	Cbr2	P08074	2	2	100	4.51E-16
RUN and FYVE domain-containing protein 1	Rufy1	Q8BIJ7	5	2	100	4.51E-16
Sphingolipid delta(4)-desaturase DES1	Degs1	O09005	5	2	100	4.51E-16
Myosin regulatory light polypeptide 9	Myl9	Q9CQ19	197	2	100	4.51E-16
Cysteine protease ATG4B	Atg4b	Q8BGE6	6	2	100	4.51E-16
ER membrane protein complex subunit 4	Emc4	Q9CZX9	5	2	100	4.51E-16
Myosin light chain 1/3, skeletal muscle isoform	Myl1	P05977	47	5	4.467	4.90E-14
Histone H4	Hist4h4; Hist1h4a; Hist1h4k; Hist1h4c; Hist1h4i; Hist1h4n; Hist1h4h; Hist1h4f; Hist1h4j; Hist1h4b; Hist1h4m; Hist1h4d; Hist2h4; LOC100862646; LOC102641229	P62806	5944	9	2.282	1.59E-08
Calumenin	Calu	O35887	11	2	2.085	4.66E-07
Splicing factor 3A subunit 2	Sf3a2	Q62203	3	2	3.035	5.77E-07
Myosin-4	Myh4	Q5SX39	291	10	2.002	4.55E-06
Histone H2A.V	H2afv	Q3THW5	5404	3	1.893	3.63E-05

Keratin. type I cytoskeletal 16	Krt16	Q9Z2K1	1231	4	1.722	2.23E-04
C-type lectin domain family 1 member B	Clec1b	Q9JL99	25	2	2.214	2.74E-04
Gamma-soluble NSF attachment protein	Napg	Q9CWZ7	3	2	2.929	3.11E-04
Cytochrome c oxidase subunit 2	COX2	P00405	606	5	1.758	4.62E-04
Core histone macro-H2A.1	H2afy	Q9QZQ8	874	17	1.707	1.17E-03
Interferon-induced transmembrane protein 2	Ifitm2	Q99J93	2	2	2.986	1.55E-03
SRSF protein kinase 2	Srpk2	O54781	17	5	1.687	2.14E-03
Histone H2A type 3	Hist3h2a	Q8BFU2	6093	2	1.664	2.42E-03
Vacuolar protein sorting-associated protein 18 homolog	Vps18	Q8R307	7	2	3.699	3.59E-03
Alpha-N-acetylneuraminide alpha-2.8-sialyltransferase	St8sia1	Q64687	4	2	1.59	4.72E-03
Histone H2B type 2-B	Hist2h2bb	Q64525	8656	7	1.622	4.80E-03
Electron transfer flavoprotein-ubiquinone oxidoreductase. mitochondrial	Etfdh	Q921G7	184	16	1.579	6.38E-03
High mobility group protein HMG-I/HMG-Y	Hmga1; Hmga1-rs1; Hmga1b	P17095	130	2	1.536	8.31E-03
Propionyl-CoA carboxylase alpha chain. mitochondrial	Pcca	Q91ZA3	7	3	2.169	1.21E-02
CD81 antigen	Cd81	P35762	34	2	1.905	1.22E-02
Complement component 1 Q subcomponent-binding protein. mitochondrial	C1qbp	O35658	270	7	1.486	1.37E-02
Integrin alpha-IIb	Itga2b	Q9QUM0	657	24	1.54	1.83E-02
Regulator of chromosome condensation	Rcc1	Q8VE37	191	10	1.467	2.04E-02
Annexin A4	Anxa4	P97429	178	10	1.447	3.26E-02
Monoacylglycerol lipase ABHD12	Abhd12	Q99LR1	26	5	1.803	3.26E-02
Platelet glycoprotein Ib beta chain	Gp1bb	P56400	104	4	1.473	4.68E-02
CD48 antigen	Cd48	P18181	84	5	1.692	4.68E-02

Table 5C
Common deregulated proteins in the bone marrow cells of mice treated with extracellular vesicles from mice irradiated with 0.1Gy and 3Gy

Protein name	UniProt ID	Gene Symbol	Group			
			**BM 0.1Gy**		**BM 3Gy**	
			**Abundance Ratio 0.1Gy vs 0Gy**	**Abundance Ratio Adj. P-Value: 0.1Gy vs 0Gy**	**Abundance Ratio 3Gy vs 0Gy**	**Abundance Ratio Adj. P-Value: 3Gy vs 0Gy**

Annexin A4	P97429	Anxa4	7.05E-01	4.25E-03	1.45E+00	3.26E-02
Carbonyl reductase [NADPH] 1	P48758	Cbr1	6.31E-01	1.39E-03	5.48E-01	2.68E-02
Chloride intracellular channel protein 4	Q9QYB1	Clic4	6.97E-01	2.25E-02	4.91E-01	4.58E-03
High mobility group protein HMG-I/HMG-Y	P17095	Hmga1; Hmga1-rs1; Hmga1b	7.70E-01	1.66E-02	1.54E+00	8.31E-03
p21-activated protein kinase-interacting protein 1	Q9DCE5	Pak1ip1	3.82E-01	1.36E-08	1.00E-02	4.51E-16
U4/U6 small nuclear ribonucleoprotein Prp31	Q8CCF0	Prpf31	7.02E-01	2.51E-02	5.16E-01	1.17E-02
TIP41-like protein	Q8BH58	Tiprl	1.00E-02	5.57E-16	1.00E-02	4.51E-16
Cysteine protease ATG4B	Q8BGE6	Atg4b	1.00E+02	5.57E-16	1.00E+02	4.51E-16
Complement component 1 Q subcomponent-binding protein, mitochondrial	O35658	C1qbp	1.55E+00	1.02E-06	1.49E+00	1.37E-02
Calumenin	O35887	Calu	1.43E+00	7.45E-03	2.09E+00	4.66E-07
Carbonyl reductase [NADPH] 2	P08074	Cbr2	1.00E+02	5.57E-16	1.00E+02	4.51E-16
CD48 antigen	P18181	Cd48	1.49E+00	6.73E-03	1.69E+00	4.68E-02
C-type lectin domain family 1 member B	Q9JL99	Clec1b	1.45E+00	4.40E-02	2.21E+00	2.74E-04
Cytochrome c oxidase subunit 2	P00405	COX2	1.49E+00	1.66E-05	1.76E+00	4.62E-04
Sphingolipid delta(4)-desaturase DES1	O09005	Degs1	1.00E+02	5.57E-16	1.00E+02	4.51E-16
Electron transfer flavoprotein-ubiquinone oxidoreductase, mitochondrial	Q921G7	Etfdh	1.45E+00	4.25E-03	1.58E+00	6.38E-03
Integrin alpha-IIb	Q9QUM0	Itga2b	1.40E+00	6.43E-04	1.54E+00	1.83E-02
Myosin-4	Q5SX39	Myh4	1.55E+00	4.23E-07	2.00E+00	4.55E-06
Myosin light chain 1/3, skeletal muscle isoform	P05977	Myl1	1.72E+00	5.59E-04	4.47E+00	4.90E-14
Myosin regulatory light polypeptide 9	Q9CQ19	Myl9	1.00E+02	5.57E-16	1.00E+02	4.51E-16
Propionyl-CoA carboxylase alpha chain, mitochondrial	Q91ZA3	Pcca	1.83E+00	2.36E-03	2.17E+00	1.21E-02
RUN and FYVE domain-containing protein 1	Q8BIJ7	Rufy1	1.00E+02	5.57E-16	1.00E+02	4.51E-16
Alpha-N-acetylneuraminide alpha-2,8-sialyltransferase	Q64687	St8sia1	1.31E+00	8.04E-03	1.59E+00	4.72E-03
Vacuolar protein sorting-associated protein 18 homolog	Q8R307	Vps18	4.87E+00	3.30E-08	3.70E+00	3.59E-03
