# Supplementary material for: The miRNA Content of Bone Marrow-Derived Extracellular Vesicles Contributes to Protein Pathway Alterations Involved in Ionising Radiation-Induced Bystander Responses
Source: Int J Mol Sci. 2023 May 11;24(10):8607. doi: 10.3390/ijms24108607 (PMC10218377; doi:10.3390/ijms24108607)
Supplement: Supplementary file 1 [file ijms-24-08607-s001.zip › Supplementary Table S6.pdf]

Supplementary Table S6. Pathways associated with significantly altered proteins in the bone marrow cells treated with bone marrow-derived extracellular vesicles of mice irradiated with 0.1Gy (A) and 3Gy (B) and common pathways associated with both treatment groups. Pathway analysis was performed with Pathdip. FDR: false discovery rate, to control FDR Benjamini–Hochberg procedure (BH-method) was used).

Table 6A
Pathways associated with deregulated proteins in the bone marrow cells of mice treated with extracellular vesicles from mice irradiated with 0.1Gy

Pathway Name	KEGG pathway class	KEGG pathway subclass	p-value	q-value (FDR: BH-method)
Oxidative phosphorylation	1. Metabolism	1.2 Energy metabolism	4.29E-07	1.21E-04
Fatty acid elongation	1. Metabolism	1.3 Lipid metabolism	3.05E-04	8.64E-03
Sphingolipid metabolism	1. Metabolism	1.3 Lipid metabolism	2.13E-03	4.02E-02
Fatty acid degradation	1. Metabolism	1.3 Lipid metabolism	2.05E-03	4.14E-02
N-Glycan biosynthesis	1. Metabolism	1.7 Glycan biosynthesis and metabolism	2.16E-04	7.63E-03
Protein processing in endoplasmic reticulum	2. Genetic information processing	2.3 Folding, sorting and degradation	2.26E-06	2.14E-04
Lysosome	4. Cellular Processes	4.1 Transport and catabolism	1.14E-06	1.62E-04
Phagosome	4. Cellular Processes	4.1 Transport and catabolism	1.48E-04	8.36E-03
Thermogenesis	5. Organismal Systems	5.10 Environmental adaptation	5.38E-04	1.27E-02
Cardiac muscle contraction	5. Organismal Systems	5.3 Circulatory system	8.45E-04	1.84E-02
Legionellosis	6.Diseases-Infectious	6.4 Infectious disease: bacterial	4.24E-05	3.00E-03
Vibrio cholerae infection	6.Diseases-Infectious	6.4 Infectious disease: bacterial	1.76E-04	7.13E-03
Alzheimer disease	6.Diseases-Infectious	6.7 Neurodegenerative disease	1.57E-04	7.43E-03
Parkinson disease	6.Diseases-Infectious	6.7 Neurodegenerative disease	2.53E-04	7.95E-03
Huntington disease	6.Diseases-Infectious	6.7 Neurodegenerative disease	4.38E-04	1.13E-02

Table 6B
Pathways associated with deregulated proteins in the bone marrow cells of mice treated with extracellular vesicles from mice irradiated with 3Gy

Pathway Name	KEGG pathway class	KEGG pathway subclass	p-value	q-value (FDR: BH-method)
Fatty acid elongation	1. Metabolism	1.3 Lipid metabolism	7.18E-03	1.54E-02
Lysine degradation	1. Metabolism	1.5 Amino acid metabolism	1.71E-13	1.39E-11
Basal transcription factors	2. Genetic information processing	2.1 Transcription	1.43E-13	1.74E-11
Ribosome	2. Genetic information processing	2.2 Translation	2.29E-08	6.96E-07
Protein processing in endoplasmic reticulum	2. Genetic information processing	2.3 Folding, sorting and degradation	4.25E-08	1.15E-06

Ubiquitin mediated proteolysis	2. Genetic information processing	2.3 Folding, sorting and degradation	3.79E-07	5.12E-06
DNA replication	2. Genetic information processing- Replication and repair	2.4 Replication and repair	8.26E-11	5.02E-09
Nucleotide excision repair	2. Genetic information processing- Replication and repair	2.4 Replication and repair	1.43E-07	3.15E-06
Homologous recombination	2. Genetic information processing- Replication and repair	2.4 Replication and repair	2.11E-07	3.41E-06
NF-kappa B signaling	3. Environmental Information Processing- Signal transduction	3.2 Signal transduction	5.17E-07	5.98E-06
ErbB signaling	3. Environmental Information Processing- Signal transduction	3.2 Signal transduction	1.19E-06	1.04E-05
PI3K-Akt signaling	3. Environmental Information Processing- Signal transduction	3.2 Signal transduction	6.53E-06	3.87E-05
JAK-STAT signaling	3. Environmental Information Processing- Signal transduction	3.2 Signal transduction	1.27E-05	6.54E-05
Hippo signaling	3. Environmental Information Processing- Signal transduction	3.2 Signal transduction	1.40E-05	6.95E-05
Sphingolipid signaling	3. Environmental Information Processing- Signal transduction	3.2 Signal transduction	2.46E-05	1.13E-04
Apelin signaling	3. Environmental Information Processing- Signal transduction	3.2 Signal transduction	3.41E-05	1.45E-04
Hedgehog signaling	3. Environmental Information Processing- Signal transduction	3.2 Signal transduction	5.02E-05	1.91E-04
TNF signaling	3. Environmental Information Processing- Signal transduction	3.2 Signal transduction	9.51E-05	3.30E-04
Ras signaling	3. Environmental Information Processing- Signal transduction	3.2 Signal transduction	1.12E-04	3.74E-04
HIF-1 signaling	3. Environmental Information Processing- Signal transduction	3.2 Signal transduction	1.98E-04	6.18E-04
MAPK signaling	3. Environmental Information Processing- Signal transduction	3.2 Signal transduction	2.33E-04	7.08E-04
Notch signaling	3. Environmental Information Processing- Signal transduction	3.2 Signal transduction	1.07E-03	2.71E-03
TGF-beta signaling	3. Environmental Information Processing- Signal transduction	3.2 Signal transduction	4.37E-03	9.92E-03
Wnt signaling	3. Environmental Information Processing- Signal transduction	3.2 Signal transduction	5.16E-03	1.16E-02
cAMP signaling	3. Environmental Information Processing- Signal transduction	3.2 Signal transduction	6.19E-03	1.37E-02

FoxO signaling	3. Environmental Information Processing-Signal transduction	3.2 Signal transduction	6.53E-03	1.43E-02
Mitophagy - animal	4. Cellular Processes	4.1 Transport and catabolism	4.57E-05	1.82E-04
Phagosome	4. Cellular Processes	4.1 Transport and catabolism	8.76E-03	1.87E-02
Oocyte meiosis	4. Cellular Processes-Cell growth and death	4.2 Cell growth and death	3.35E-05	1.45E-04
Cell cycle	4. Cellular Processes-Cell growth and death	4.2 Cell growth and death	9.60E-04	2.46E-03
Necroptosis	4. Cellular Processes-Cell growth and death	4.2 Cell growth and death	1.41E-07	3.43E-06
p53 signaling	4. Cellular Processes-Cell growth and death	4.2 Cell growth and death	6.23E-07	6.88E-06
Apoptosis	4. Cellular Processes-Cell growth and death	4.2 Cell growth and death	2.44E-04	7.23E-04
Cellular senescence	4. Cellular Processes-Cell growth and death	4.2 Cell growth and death	3.53E-03	8.16E-03
Focal adhesion	4. Cellular Processes	4.3 Cellular community - eukaryotes	2.16E-06	1.64E-05
Adherens junction	4. Cellular Processes	4.3 Cellular community - eukaryotes	1.94E-04	6.13E-04
Tight junction	4. Cellular Processes	4.3 Cellular community - eukaryotes	4.71E-06	3.01E-05
Gap junction	4. Cellular Processes	4.3 Cellular community - eukaryotes	2.91E-04	8.52E-04
Antigen processing and presentation	5. Organismal Systems-Immune system	5.1 Immune system	1.22E-10	5.92E-09
Fc epsilon RI signaling	5. Organismal Systems-Immune system	5.1 Immune system	1.54E-07	3.11E-06
Cytosolic DNA-sensing	5. Organismal Systems-Immune system	5.1 Immune system	2.33E-07	3.55E-06
B cell receptor signaling	5. Organismal Systems-Immune system	5.1 Immune system	3.66E-06	2.54E-05
C-type lectin receptor signaling	5. Organismal Systems-Immune system	5.1 Immune system	4.86E-06	3.03E-05
RIG-I-like receptor signaling	5. Organismal Systems-Immune system	5.1 Immune system	1.02E-05	5.78E-05
Toll-like receptor signaling	5. Organismal Systems-Immune system	5.1 Immune system	1.11E-05	6.02E-05
NOD-like receptor signaling	5. Organismal Systems-Immune system	5.1 Immune system	1.28E-05	6.50E-05
Chemokine signaling	5. Organismal Systems-Immune system	5.1 Immune system	2.80E-05	1.24E-04
Th1 and Th2 cell differentiation	5. Organismal Systems-Immune system	5.1 Immune system	3.41E-05	1.45E-04
T cell receptor signaling	5. Organismal Systems-Immune system	5.1 Immune system	5.01E-05	1.93E-04
IL-17 signaling	5. Organismal Systems-Immune system	5.1 Immune system	9.02E-05	3.18E-04
Th17 cell differentiation	5. Organismal Systems-Immune system	5.1 Immune system	7.14E-04	1.87E-03
Complement and coagulation cascades	5. Organismal Systems-Immune system	5.1 Immune system	6.55E-03	1.42E-02
Progesterone-mediated oocyte maturation	5. Organismal Systems	5.2 Endocrine system	1.38E-04	4.52E-04
Parathyroid hormone synthesis, secretion and action	5. Organismal Systems	5.2 Endocrine system	9.94E-05	3.40E-04
Prolactin signaling	5. Organismal Systems	5.2 Endocrine system	1.82E-04	5.90E-04

Oxytocin signaling	5. Organismal Systems	5.2 Endocrine system	1.85E-04	5.90E-04
GnRH signaling	5. Organismal Systems	5.2 Endocrine system	4.73E-04	1.34E-03
Insulin signaling	5. Organismal Systems	5.2 Endocrine system	7.03E-04	1.86E-03
Adipocytokine signaling	5. Organismal Systems	5.2 Endocrine system	7.99E-04	2.07E-03
Estrogen signaling	5. Organismal Systems	5.2 Endocrine system	1.27E-03	3.13E-03
Thyroid hormone signaling	5. Organismal Systems	5.2 Endocrine system	1.98E-03	4.67E-03
Relaxin signaling	5. Organismal Systems	5.2 Endocrine system	2.02E-03	4.73E-03
Neurotrophin signaling	5. Organismal Systems	5.6 Nervous system	9.07E-07	8.82E-06
Osteoclast differentiation	5. Organismal Systems	5.8 Development and regeneration	1.25E-06	1.04E-05
Transcriptional misregulation in cancer	6.Diseases-cancer	6.1 Cancer: overview	7.08E-07	7.48E-06
Viral carcinogenesis	6.Diseases-cancer	6.1 Cancer: overview	8.02E-07	8.12E-06
Central carbon metabolism in cancer	6.Diseases-cancer	6.1 Cancer: overview	6.37E-05	2.34E-04
Proteoglycans in cancer	6.Diseases-cancer	6.1 Cancer: overview	4.20E-04	1.22E-03
Pathways in cancer	6.Diseases-cancer	6.1 Cancer: overview	1.95E-02	4.09E-02
MicroRNAs in cancer	6.Diseases-cancer	6.1 Cancer: overview	1.12E-05	5.92E-05
Choline metabolism in cancer	6.Diseases-cancer	6.1 Cancer: overview	2.76E-05	1.24E-04
Type II diabetes mellitus	6.Diseases	6.10 Endocrine and metabolic disease	1.11E-04	3.73E-04
Insulin resistance	6.Diseases	6.10 Endocrine and metabolic disease	4.28E-04	1.22E-03
AGE-RAGE signaling pathway in diabetic complications	6.Diseases	6.10 Endocrine and metabolic disease	6.62E-04	1.79E-03
Cushing syndrome	6.Diseases	6.10 Endocrine and metabolic disease	1.45E-03	3.52E-03
Non-small cell lung cancer	6.Diseases-cancer	6.2 Cancer: specific types	1.57E-07	2.93E-06
Acute myeloid leukemia	6.Diseases-cancer	6.2 Cancer: specific types	3.78E-06	2.55E-05
Small cell lung cancer	6.Diseases-cancer	6.2 Cancer: specific types	4.38E-06	2.88E-05
Glioma	6.Diseases-cancer	6.2 Cancer: specific types	1.09E-05	6.03E-05
Renal cell carcinoma	6.Diseases-cancer	6.2 Cancer: specific types	2.16E-05	1.03E-04
Chronic myeloid leukemia	6.Diseases-cancer	6.2 Cancer: specific types	6.17E-05	2.31E-04
Endometrial cancer	6.Diseases-cancer	6.2 Cancer: specific types	7.76E-05	2.82E-04
Pancreatic cancer	6.Diseases-cancer	6.2 Cancer: specific types	6.19E-04	1.69E-03
Colorectal cancer	6.Diseases-cancer	6.2 Cancer: specific types	1.69E-03	4.03E-03
Prostate cancer	6.Diseases-cancer	6.2 Cancer: specific types	3.66E-03	8.38E-03
Bladder cancer	6.Diseases-cancer	6.2 Cancer: specific types	4.30E-05	1.74E-04
Thyroid cancer	6.Diseases-cancer	6.2 Cancer: specific types	2.39E-04	7.16E-04
Gastric cancer	6.Diseases-cancer	6.2 Cancer: specific types	1.09E-03	2.72E-03
Hepatocellular carcinoma	6.Diseases-cancer	6.2 Cancer: specific types	1.13E-03	2.79E-03
Breast cancer	6.Diseases-cancer	6.2 Cancer: specific types	5.48E-03	1.22E-02
Melanoma	6.Diseases-cancer	6.2 Cancer: specific types	9.01E-03	1.90E-02
Hepatitis C	6.Diseases-Infectious	6.3 Infectious disease: viral	4.90E-07	5.95E-06
Hepatitis B	6.Diseases-Infectious	6.3 Infectious disease: viral	8.13E-05	2.90E-04

Human immunodeficiency virus 1 infection	6.Diseases-Infectious	6.3 Infectious disease: viral	9.14E-07	8.55E-06
Herpes simplex infection	6.Diseases-Infectious	6.3 Infectious disease: viral	1.10E-06	9.87E-06
Measles	6.Diseases-Infectious	6.3 Infectious disease: viral	1.45E-06	1.17E-05
Influenza A	6.Diseases-Infectious	6.3 Infectious disease: viral	6.84E-06	3.95E-05
Epstein-Barr virus infection	6.Diseases-Infectious	6.3 Infectious disease: viral	4.67E-05	1.83E-04
Human papillomavirus infection	6.Diseases-Infectious	6.3 Infectious disease: viral	2.18E-04	6.69E-04
Human cytomegalovirus infection	6.Diseases-Infectious	6.3 Infectious disease: viral	4.97E-04	1.39E-03
Kaposi sarcoma-associated herpesvirus infection	6.Diseases-Infectious	6.3 Infectious disease: viral	5.28E-04	1.46E-03
Human T-cell leukemia virus 1 infection	6.Diseases-Infectious	6.3 Infectious disease: viral	6.67E-04	1.78E-03
Legionellosis	6.Diseases-Infectious	6.4 Infectious disease: bacterial	4.67E-09	1.62E-07
Pertussis	6.Diseases-Infectious	6.4 Infectious disease: bacterial	1.87E-07	3.24E-06
Pathogenic Escherichia coli infection	6.Diseases-Infectious	6.4 Infectious disease: bacterial	2.72E-07	3.89E-06
Salmonella infection	6.Diseases-Infectious	6.4 Infectious disease: bacterial	2.58E-06	1.90E-05
Shigellosis	6.Diseases-Infectious	6.4 Infectious disease: bacterial	2.82E-06	2.02E-05
Epithelial cell signaling in Helicobacter pylori infection	6.Diseases-Infectious	6.4 Infectious disease: bacterial	1.42E-05	6.88E-05
Tuberculosis	6.Diseases-Infectious	6.4 Infectious disease: bacterial	3.51E-05	1.44E-04
Bacterial invasion of epithelial cells	6.Diseases-Infectious	6.4 Infectious disease: bacterial	2.23E-02	4.63E-02
Leishmaniasis	6.Diseases-Infectious	6.5 Infectious disease: parasitic	4.29E-07	5.49E-06
Toxoplasmosis	6.Diseases-Infectious	6.5 Infectious disease: parasitic	2.28E-05	1.06E-04
Chagas disease (American trypanosomiasis)	6.Diseases-Infectious	6.5 Infectious disease: parasitic	1.50E-03	3.61E-03
Systemic lupus erythematosus	6.Diseases	6.6 Immune disease	3.17E-16	7.71E-14
Inflammatory bowel disease (IBD)	6.Diseases	6.6 Immune disease	1.67E-06	1.31E-05
Alcoholism	6.Diseases	6.8 Substance dependence	1.42E-10	5.75E-09
Fluid shear stress and atherosclerosis	6.Diseases	6.9 Cardiovascular disease	5.27E-06	3.20E-05

Table 6C

Common pathways associated with deregulated proteins in the bone marrow cells of mice treated with extracellular vesicles from mice irradiated with 0.1Gy and 3Gy

**Pathway Name**	**KEGG pathway class**	**KEGG pathway subclass**	**Group**

			BM+0.1Gy EV		BM+3Gy EV	
			**p-value**	**q-value (FDR: BH-method)**	**p-value**	**q-value (FDR: BH-method)**
Fatty acid elongation	1. Metabolism	1.3 Lipid metabolism	3.05E-04	8.64E-03	7.18E-03	1.54E-02
Protein processing in endoplasmic reticulum	2. Genetic information processing	2.3 Folding, sorting and degradation	2.26E-06	2.14E-04	4.25E-08	1.15E-06
Phagosome	4. Cellular Processes	4.1 Transport and catabolism	1.48E-04	8.36E-03	8.76E-03	1.87E-02
Legionellosis	6.Diseases-Infectious	6.4 Infectious disease: bacterial	4.24E-05	3.00E-03	4.67E-09	1.62E-07
