# Supplementary material for: The miRNA Content of Bone Marrow-Derived Extracellular Vesicles Contributes to Protein Pathway Alterations Involved in Ionising Radiation-Induced Bystander Responses
Source: Int J Mol Sci. 2023 May 11;24(10):8607. doi: 10.3390/ijms24108607 (PMC10218377; doi:10.3390/ijms24108607)
Supplement: Supplementary file 1 [file ijms-24-08607-s001.zip › Supplementary Table S7.pdf]

Supplementary Table S7. Clustering of significantly deregulated proteins. Clustering was performed with STRING using MCL algorithm. Clusters with less than 5 proteins are not presented, except in group BM+3Gy EV, where the input protein number was significantly lower than in the other three treatment groups.

	Proteins	Related GO biological processes
	**BM 0.1Gy**	
cluster 1	Atp5d, Atp5h, Atp6v1g1, Cox6b1, Gm11273, Hint1, Ndufab1, Uqcrh	Mitochondria-related biological processes
cluster 2	Ccdc124, Eef1b2, Eef1d, Oas1a, Rplp1, Rps12,Rps15	Translational regulation and nitrogen biosynthetic processes
cluster 3	Ahsg, Itih2, Plg, Timp3, Tmcc1, Vapa	Endopeptidase inhibitor activity
cluster4	Abcd3, Cat, Cbr1, Gstm5, Mgst1	Cellular detoxification and oxidation-reduction processes
	**BM 3Gy**	
cluster 1	Ass1, Dtd1, Msh6, Ncapg2, Pold2, Prim1, Ptma, Rfc4, Rfc5, Tk1, Wdhd1, Wdr48	DNA damage response, DNA repair, cellular stress-response, biosynthetic and metabolic processes
cluster 2	Cnot7, Dis3, Exosc2, Exosc9, Gtpbp4, Pak1ip1,Parn, Rbm27, Rrp1, Rsl1d1, Skiv2l2, Wdr43	RNA synthesis, processing and metabolism
cluster 3	Agfg1, Appl1, Baz1a, Ctbp2, Ctcf, Hdac2,Smarcb1, Smarce1, Tbl1xr1, Ubtf	Chromatin organization, remodeling, histone modifications, transcriptional regulation (mRNA, miRNA)
cluster 4	Atp5j, Atp6ap2, Bcs1l, Hint1, Ndufa11, Ndufb9, Ndufs4, mt-Co1, mt-Nd4	Mitochondria-related biological processes
cluster 5	Aqr, Edc4, Lsm2, Lsm5, Ppwd1, Prpf3, Prpf31, Prpf6	mRNA processing, splicing
cluster 6	Add2, Car1, Car2, Epb4.2, Slc4a1, Spta1, Tmod1	Hemopoiesis, actin filament capping, cellular homeostasis, one-carbon metabolic process, organ development
cluster 7	Ccdc124, Rpl18, Rpl35, Rpl36al, Rps15, Rps28, Ufsp2	Translation, ribosoma-related biological processes
cluster 8	Eed, Fmnl3, Lamtor1, Mvp, Naa25, Psip1	regulation of intracellular signal transduction (TOR signaling), cellular response to amino acid stimulus, protein modification
cluster 9	Cpsf3, Cstf1, Cstf2, Cstf3, Lactb, Ssu72	mRNA processing, mRNA 3'-end processing
cluster 10	Bola1, Erlin1, Fxn, Iba57, Nfs1	Metabolic and biosynthetic processes, cellular component biogenesis (Iron-sulfur cluster)
	**BM+0.1Gy EV**	
cluster 1	Apool, Atp5l, Cisd1, Cyc1, Hypk, Ndufv1,Uqcrb, Uqcrc2, Uqcrh, mt-Co2	Mitochondria-related biological processes
cluster 2	C1qbp, Cd14, Cd38, Cd48, Clec1b, Gp9, Itga1, Itga2b, Itgb3, Pf4	Cellular response to stress, signal transduction, immune system processes, blood coagulation-related processes
cluster 3	Dis3, Pak1ip1, Prpf31, Rps11, Rps14	Ribonucleoprotein complex biogenesis
cluster 4	Ctsa, Ctse, Ctsh, Glb1, St8sia1	catabolic and metabolic processes, glycosylation, response to thyroid hormone
	**BM+3Gy EV**	
cluster 1	H2afv, H2afy, Hist1h4j, Hist2h2bb, Hist3h2a, Rcc1, Rtf1	Chromatin organization, remodeling, silencing, histone modifications
cluster 2	Cd48, Clec1b, Fcer1g, Itga2b	immune response, cellular response to stress, signal transduction

cluster 3	Pak1ip1,Prpf31,Sf3a2	Spliceosomal complex (U2-type precatalytic spliceosome, spliceosomal snRNP complex)
cluster 4	Myh4,Myl1,Myl9	Myosin complex, Myofibril
cluster 5	Anxa4,Cd81,Ifitm2	Immune response, metabolic processes
