# Supplementary material for: The miRNA Content of Bone Marrow-Derived Extracellular Vesicles Contributes to Protein Pathway Alterations Involved in Ionising Radiation-Induced Bystander Responses
Source: Int J Mol Sci. 2023 May 11;24(10):8607. doi: 10.3390/ijms24108607 (PMC10218377; doi:10.3390/ijms24108607)
Supplement: Supplementary file 1 [file ijms-24-08607-s001.zip › Supplementary Table S8.pdf]

Supplementary Table S8. Common significantly altered proteins and associated pathways in the bone marrow cells either irradiated with 0.1Gy (A) or 3Gy (B) or treated with bone marrow-derived extracellular vesicles of mice irradiated with 0.1Gy (A) or 3Gy (B). Pathway analysis was performed with Pathdip and Benjamini–Hochberg procedure (BH-method) was used to control FDR. FDR: false discovery rate

Table 8A-
Common proteins and pathways between BM 0.1Gy and BM+0.1Gy EV

Common proteins
(BM 0.1Gy and BM+0.1Gy EV)

Protein name	UniProt ID	Gene Symbol	Group: BM 0.1Gy Abundance Ratio	Group: BM 0.1Gy Abundance Ratio Adj. P-Value	Group: BM+0.1Gy EV Abundance Ratio	Group: BM+0.1Gy EV Abundance Ratio Adj. P-Value
Peroxiredoxin-4	O08807	Prdx4	2.36E+00	3.46E-02	1.51E+00	1.11E-03
Sphingolipid delta(4)-desaturase DES1	O09005	Degs1	1.00E-02	2.23E-16	1.00E+02	5.57E-16
Fatty acid-binding protein, adipocyte	P04117	Fabp4	1.77E-01	1.12E-07	7.21E-01	4.64E-02
Beta-galactosidase	P23780	Glb1	1.00E-02	2.23E-16	4.81E-01	3.20E-06
Carbonyl reductase [NADPH] 1	P48758	Cbr1	2.51E+00	3.14E-02	6.31E-01	1.39E-03
RNA-binding protein FUS	P56959	Fus	5.33E+00	4.45E-06	6.44E-01	2.05E-03
Cathepsin E	P70269	Ctse	1.09E+01	2.23E-16	1.33E+00	2.79E-02
Cytochrome b-c1 complex subunit 6, mitochondrial	P99028	Uqcrh	3.51E+00	2.11E-03	1.72E+00	2.29E-04
FAS-associated factor 2	Q3TDN2	Faf2	1.00E+02	2.23E-16	1.00E-02	5.57E-16
RUN and FYVE domain-containing protein 1	Q8BIJ7	Rufy1	1.00E-02	2.23E-16	1.00E+02	5.57E-16
N-acetylglucosamine-1-phosphodiester alpha-N-acetylglucosaminidase	Q8BJ48	Nagpa	1.00E+02	2.23E-16	1.97E+00	6.56E-04
Mitochondrial 2-oxodicarboxylate carrier	Q8BZ09	Slc25a21	3.47E+00	2.10E-02	1.80E+00	2.60E-03
Thioredoxin domain-containing protein 5	Q91W90	Txndc5	2.25E+00	2.10E-02	1.35E+00	1.12E-02
Keratin, type II cuticular Hb4	Q99M73	Krt84	3.34E+00	3.52E-02	1.85E+00	5.77E-05
Exosome complex exonuclease RRP44	Q9CSH3	Dis3	1.00E-02	2.23E-16	5.50E-01	5.38E-05
Sorting nexin-29	Q9D3S3	Snx29	1.00E+02	2.23E-16	1.82E-01	5.57E-16
Trans-2-enoyl-CoA reductase, mitochondrial	Q9DCS3	Mecr	8.05E+00	3.53E-06	1.00E+02	5.57E-16

Mitochondrial import inner membrane translocase subunit Tim8 A	Q9WVA2	Timm8a1	1.00E+02	2.23E-16	1.63E+00	2.34E-03

Common pathways
(BM 0.1Gy and BM+0.1Gy EV)

Pathway Name	KEGG pathway class	KEGG pathway subclass	Group			
			BM 0.1Gy		BM+3Gy EV	
			p-value	q-value (FDR: BH-method)	p-value	q-value (FDR: BH-method)
Oxidative phosphorylation	1. Metabolism	1.2 Energy metabolism	3.74E-05	9.16E-04	4.29E-07	1.21E-04
Protein processing in endoplasmic reticulum	2. Genetic information processing	2.3 Folding, sorting and degradation	7.04E-05	1.09E-03	2.26E-06	2.14E-04
Lysosome	4. Cellular Processes	4.1 Transport and catabolism	3.50E-04	3.32E-03	1.14E-06	1.62E-04
Phagosome	4. Cellular Processes	4.1 Transport and catabolism	1.21E-04	1.69E-03	1.48E-04	8.36E-03
Legionellosis	6.Diseases-Infectious	6.4 Infectious disease: bacterial	2.66E-05	7.83E-04	4.24E-05	3.00E-03
Huntington disease	6.Diseases	6.7 Neurodegenerative disease	1.29E-03	7.59E-03	4.38E-04	1.13E-02
Parkinson disease	6.Diseases	6.7 Neurodegenerative disease	2.57E-04	2.79E-03	2.53E-04	7.95E-03

Table 8B-
Common proteins and pathways between BM 3Gy and BM+3Gy EV

Common proteins
(BM 3Gy and BM+3Gy EV)

Protein name	UniProt ID	Gene Symbol	Group			
			BM 3Gy		BM+3Gy EV	
			Abundance Ratio	Abundance Ratio Adj. P-Value	Abundance Ratio	Abundance Ratio Adj. P-Value
Sphingolipid delta(4)-desaturase DES1 OS	O09005	Degs1	1.00E-02	6.12E-17	1.00E+02	4.51E-16
SRSF protein kinase 2 OS	O54781	Srpk2	2.53E+01	3.94E-11	1.69E+00	2.14E-03
Carbonic anhydrase 2 OS	P00920	Car2	2.03E+01	5.19E-11	6.44E-01	3.18E-02
Hemoglobin subunit alpha OS	P01942	Hba-a1; Hba-a2	3.80E+01	1.36E-15	6.53E-01	4.33E-02

Hemoglobin subunit beta-1 OS	P02088	Hbb-b1; Beta-s; Hbb-bs; Hbb-bt	5.17E+01	6.12E-17	5.76E-01	1.80E-03
Hemoglobin subunit beta-2 OS	P02089	Hbb-b2	6.02E+01	6.12E-17	5.94E-01	4.19E-03
Carbonyl reductase [NADPH] 2 OS	P08074	Cbr2	1.00E-02	6.12E-17	1.00E+02	4.51E-16
Carbonic anhydrase 1 OS	P13634	Car1	3.15E+01	3.50E-14	5.94E-01	4.30E-03
Bisphosphoglycerate mutase OS	P15327	Bpgm	4.91E+00	1.36E-03	4.80E-01	4.34E-07
Carbonyl reductase [NADPH] 1 OS	P48758	Cbr1	3.43E+00	2.77E-02	5.48E-01	2.68E-02
Platelet glycoprotein Ib beta chain OS	P56400	Gp1bb	3.38E+00	2.32E-02	1.47E+00	4.68E-02
Glycogen synthase kinase-3 alpha OS	Q2NL51	Gsk3a	8.14E+01	6.12E-17	4.82E-01	4.60E-06
Nesprin-3 OS	Q4FZC9	4831426I19Rik; Syne3	1.00E-02	6.12E-17	1.00E-02	4.51E-16
RUN and FYVE domain-containing protein 1 OS	Q8BIJ7	Rufy1	1.00E-02	6.12E-17	1.00E+02	4.51E-16
U4/U6 small nuclear ribonucleoprotein Prp31 OS	Q8CCF0	Prpf31	9.58E+00	6.04E-06	5.16E-01	1.17E-02
Vacuolar protein sorting-associated protein 18 homolog OS	Q8R307	Vps18	1.00E-02	6.12E-17	3.70E+00	3.59E-03
Ubiquitin-associated domain-containing protein 1 OS	Q8VDI7	Ubac1	4.42E+00	2.34E-02	3.29E-01	4.97E-03
Galectin-related protein OS	Q8VED9	Lgalsl	1.00E-02	6.12E-17	2.24E-01	2.18E-05
Propionyl-CoA carboxylase alpha chain, mitochondrial OS	Q91ZA3	Pcca	1.00E-02	6.12E-17	2.17E+00	1.21E-02
Interferon-induced transmembrane protein 2 OS	Q99J93	Ifitm2	1.00E-02	6.12E-17	2.99E+00	1.55E-03
N-acylneuraminate-9-phosphatase OS	Q9CPT3	Nanp	1.00E+02	6.12E-17	3.31E-01	5.32E-03
Myosin regulatory light polypeptide 9 OS	Q9CQ19	Myl9	1.00E+02	6.12E-17	1.00E+02	4.51E-16
Gamma-soluble NSF attachment protein OS	Q9CWZ7	Napg	1.00E-02	6.12E-17	2.93E+00	3.11E-04
Alpha-hemoglobin-stabilizing protein	Q9CY02	Ahsp	3.80E+00	1.02E-02	4.64E-01	6.87E-07
p21-activated protein kinase-interacting protein 1	Q9DCE5	Pak1ip1	1.00E-02	6.12E-17	1.00E-02	4.51E-16
Integrin alpha-IIb	Q9QUM0	Itga2b	3.72E+00	1.18E-02	1.54E+00	1.83E-02

Common pathways
(BM 3Gy and BM+3Gy EV)

Pathway Name	KEGG pathway class	KEGG pathway subclass	Group	
			BM 0.1Gy	BM+3Gy EV

			p-value	q-value (FDR: BH-method)	p-value	q-value (FDR: BH-method)
Basal transcription factors	2. Genetic information processing	2.1 Transcription	3.53E-04	3.29E-03	1.43E-13	1.74E-11
Ribosome	2. Genetic information processing	2.2 Translation	1.47E-03	9.79E-03	2.29E-08	6.96E-07
Ubiquitin mediated proteolysis	2. Genetic information processing	2.3 Folding, sorting and degradation	3.52E-04	3.38E-03	3.79E-07	5.12E-06
DNA replication	2. Genetic information processing- Replication and repair	2.4 Replication and repair	1.96E-09	1.00E-07	8.26E-11	5.02E-09
Homologous recombination	2. Genetic information processing- Replication and repair	2.4 Replication and repair	4.21E-11	6.46E-09	2.11E-07	3.41E-06
Nucleotide excision repair	2. Genetic information processing- Replication and repair	2.4 Replication and repair	1.25E-06	2.55E-05	1.43E-07	3.15E-06
ErbB signaling	3. Environmental Information Processing- Signal tranduction	3.2 Signal transduction	9.23E-04	7.27E-03	1.19E-06	1.04E-05
HIF-1 signaling	3. Environmental Information Processing- Signal tranduction	3.2 Signal transduction	2.83E-03	1.47E-02	1.98E-04	6.18E-04
Hippo signaling	3. Environmental Information Processing- Signal tranduction	3.2 Signal transduction	5.18E-04	4.42E-03	1.40E-05	6.95E-05
NF-kappa B signaling	3. Environmental Information Processing- Signal tranduction	3.2 Signal transduction	9.71E-04	7.45E-03	5.17E-07	5.98E-06
PI3K-Akt signaling	3. Environmental Information Processing- Signal tranduction	3.2 Signal transduction	1.33E-05	2.04E-04	6.53E-06	3.87E-05
Sphingolipid signaling	3. Environmental Information Processing- Signal tranduction	3.2 Signal transduction	1.01E-02	3.72E-02	2.46E-05	1.13E-04
TNF signaling	3. Environmental Information Processing- Signal tranduction	3.2 Signal transduction	5.05E-03	2.28E-02	9.51E-05	3.30E-04
Mitophagy - animal	4. Cellular Processes	4.1 Transport and catabolism	2.52E-03	1.38E-02	4.57E-05	1.82E-04

Apoptosis	4. Cellular Processes-Cell growth and death	4.2 Cell growth and death	2.54E-06	4.59E-05	2.44E-04	7.23E-04
Cell cycle	4. Cellular Processes-Cell growth and death	4.2 Cell growth and death	4.65E-11	4.75E-09	9.60E-04	2.46E-03
Cellular senescence	4. Cellular Processes-Cell growth and death	4.2 Cell growth and death	3.18E-03	1.60E-02	3.53E-03	8.16E-03
Oocyte meiosis	4. Cellular Processes-Cell growth and death	4.2 Cell growth and death	6.08E-08	1.44E-06	3.35E-05	1.45E-04
p53 signaling	4. Cellular Processes-Cell growth and death	4.2 Cell growth and death	1.14E-03	8.14E-03	6.23E-07	6.88E-06
Tight junction	4. Cellular Processes	4.3 Cellular community - eukaryotes	3.25E-04	3.22E-03	4.71E-06	3.01E-05
B cell receptor signaling	5. Organismal Systems-Immune system	5.1 Immune system	3.50E-03	1.71E-02	3.66E-06	2.54E-05
IL-17 signaling	5. Organismal Systems-Immune system	5.1 Immune system	4.20E-03	1.98E-02	9.02E-05	3.18E-04
NOD-like receptor signaling	5. Organismal Systems-Immune system	5.1 Immune system	2.50E-03	1.40E-02	1.28E-05	6.50E-05
T cell receptor signaling	5. Organismal Systems-Immune system	5.1 Immune system	6.59E-03	2.73E-02	5.01E-05	1.93E-04
Thyroid hormone signaling	5. Organismal Systems	5.2 Endocrine system	4.36E-03	2.00E-02	1.98E-03	4.67E-03
Neurotrophin signaling	5. Organismal Systems	5.6 Nervous system	1.82E-06	3.48E-05	9.07E-07	8.82E-06
Osteoclast differentiation	5. Organismal Systems	5.8 Development and regeneration	7.84E-03	3.13E-02	1.25E-06	1.04E-05
Central carbon metabolism in cancer	6.Diseases-cancer	6.1 Cancer: overview	1.01E-03	7.53E-03	6.37E-05	2.34E-04
MicroRNAs in cancer	6.Diseases-cancer	6.1 Cancer: overview	3.17E-04	3.24E-03	1.12E-05	5.92E-05
Pathways in cancer	6.Diseases-cancer	6.1 Cancer: overview	1.05E-02	3.83E-02	1.95E-02	4.09E-02
Proteoglycans in cancer	6.Diseases-cancer	6.1 Cancer: overview	1.97E-03	1.18E-02	4.20E-04	1.22E-03
Viral carcinogenesis	6.Diseases-cancer	6.1 Cancer: overview	1.97E-08	6.72E-07	8.02E-07	8.12E-06
AGE-RAGE signaling pathway in diabetic complications	6.Diseases	6.10 Endocrine and metabolic disease	5.30E-03	2.36E-02	6.62E-04	1.79E-03
Acute myeloid leukemia	6.Diseases-cancer	6.2 Cancer: specific types	1.19E-02	4.26E-02	3.78E-06	2.55E-05
Colorectal cancer	6.Diseases-cancer	6.2 Cancer: specific types	2.47E-03	1.41E-02	1.69E-03	4.03E-03
Renal cell carcinoma	6.Diseases-cancer	6.2 Cancer: specific types	4.27E-04	3.75E-03	2.16E-05	1.03E-04

Thyroid cancer	6.Diseases-cancer	6.2 Cancer: specific types	2.03E-04	2.15E-03	2.39E-04	7.16E-04
Hepatitis B	6.Diseases-Infectious	6.3 Infectious disease: viral	5.38E-05	6.36E-04	8.13E-05	2.90E-04
Hepatitis C	6.Diseases-Infectious	6.3 Infectious disease: viral	5.25E-08	1.34E-06	4.90E-07	5.95E-06
Herpes simplex infection	6.Diseases-Infectious	6.3 Infectious disease: viral	1.88E-05	2.62E-04	1.10E-06	9.87E-06
Human cytomegalovirus infection	6.Diseases-Infectious	6.3 Infectious disease: viral	3.43E-03	1.70E-02	4.97E-04	1.39E-03
Human immunodeficiency virus 1 infection	6.Diseases-Infectious	6.3 Infectious disease: viral	1.18E-03	8.21E-03	9.14E-07	8.55E-06
Human T-cell leukemia virus 1 infection	6.Diseases-Infectious	6.3 Infectious disease: viral	1.73E-03	1.11E-02	6.67E-04	1.78E-03
Influenza A	6.Diseases-Infectious	6.3 Infectious disease: viral	2.24E-03	1.30E-02	6.84E-06	3.95E-05
Kaposi sarcoma-associated herpesvirus infection	6.Diseases-Infectious	6.3 Infectious disease: viral	9.71E-03	3.63E-02	5.28E-04	1.46E-03
Bacterial invasion of epithelial cells	6.Diseases-Infectious	6.4 Infectious disease: bacterial	1.80E-03	1.12E-02	2.23E-02	4.63E-02
Legionellosis	6.Diseases-Infectious	6.4 Infectious disease: bacterial	2.22E-03	1.31E-02	4.67E-09	1.62E-07
Pathogenic Escherichia coli infection	6.Diseases-Infectious	6.4 Infectious disease: bacterial	5.57E-06	9.50E-05	2.72E-07	3.89E-06
Pertussis	6.Diseases-Infectious	6.4 Infectious disease: bacterial	7.81E-04	6.31E-03	1.87E-07	3.24E-06
Salmonella infection	6.Diseases-Infectious	6.4 Infectious disease: bacterial	1.93E-03	1.18E-02	2.58E-06	1.90E-05
Shigellosis	6.Diseases-Infectious	6.4 Infectious disease: bacterial	8.38E-03	3.21E-02	2.82E-06	2.02E-05
