# Supplementary material for: The miRNA Content of Bone Marrow-Derived Extracellular Vesicles Contributes to Protein Pathway Alterations Involved in Ionising Radiation-Induced Bystander Responses
Source: Int J Mol Sci. 2023 May 11;24(10):8607. doi: 10.3390/ijms24108607 (PMC10218377; doi:10.3390/ijms24108607)
Supplement: Supplementary file 1 [file ijms-24-08607-s001.zip › Supplementary Table S9.pdf]

Supplementary Table S9. Common pathways between differentially expressed miRNAs from bone marrow-derived extracellular vesicles from mice irradiated with 3Gy and deregulated proteins in bone marrow cells treated with bone marrow-derived extracellular vesicles of mice irradiated with 3Gy.

**Common pathways between 3Gy EV miRNAs and BM+3Gy EV proteins**		
**Pathway Name**	**KEGG pathway class**	**KEGG pathway subclass**
Lysine degradation	1. Metabolism	1.5 Amino acid metabolism
Protein processing in endoplasmic reticulum	2. Genetic information processing	2.3 Folding, sorting and degradation
Focal adhesion	4. Cellular Processes	4.3 Cellular community - eukaryotes
Adherens junction	4. Cellular Processes	4.3 Cellular community - eukaryotes
Cell cycle	4. Cellular Processes- Cell growth and death	4.2 Cell growth and death
Progesterone-mediated oocyte maturation	5. Organismal Systems	5.2 Endocrine system
Proteoglycans in cancer	6.Diseases-cancer	6.1 Cancer: overview
Prostate cancer	6.Diseases-cancer	6.2 Cancer: specific types
Colorectal cancer	6.Diseases-cancer	6.2 Cancer: specific types
Glioma	6.Diseases-cancer	6.2 Cancer: specific types
Chronic myeloid leukemia	6.Diseases-cancer	6.2 Cancer: specific types
Pathways in cancer	6.Diseases-cancer	6.1 Cancer: overview
Pancreatic cancer	6.Diseases-cancer	6.2 Cancer: specific types
Acute myeloid leukemia	6.Diseases-cancer	6.2 Cancer: specific types
Non-small cell lung cancer	6.Diseases-cancer	6.2 Cancer: specific types
Transcriptional misregulation in cancer	6.Diseases-cancer	6.1 Cancer: overview
Hepatitis B	6.Diseases-Infectious	6.3 Infectious disease: viral
